# The role of m6A modification in the biological functions and diseases

**DOI:** 10.1038/s41392-020-00450-x

**Published:** 2021-02-21

**Authors:** Xiulin Jiang, Baiyang Liu, Zhi Nie, Lincan Duan, Qiuxia Xiong, Zhixian Jin, Cuiping Yang, Yongbin Chen

**Affiliations:** 1 Key Laboratory of Animal Models and Human Disease Mechanisms of Chinese Academy of Sciences & Yunnan Province, Kunming Institute of Zoology, 650223 Kunming, Yunnan China; 2 Kunming College of Life Science, University of Chinese Academy of Sciences, 100049 Beijing, China; 3 Kunming Medical University, 650500 Kunming, China; 4 Center for Excellence in Animal Evolution and Genetics, Chinese Academy of Sciences, 650223 Kunming, Yunnan China

**Keywords:** Cancer, Molecular biology

## Abstract

N^6^-methyladenosine (m6A) is the most prevalent, abundant and conserved internal cotranscriptional modification in eukaryotic RNAs, especially within higher eukaryotic cells. m6A modification is modified by the m6A methyltransferases, or writers, such as METTL3/14/16, RBM15/15B, ZC3H3, VIRMA, CBLL1, WTAP, and KIAA1429, and, removed by the demethylases, or erasers, including FTO and ALKBH5. It is recognized by m6A-binding proteins YTHDF1/2/3, YTHDC1/2 IGF2BP1/2/3 and HNRNPA2B1, also known as “readers”. Recent studies have shown that m6A RNA modification plays essential role in both physiological and pathological conditions, especially in the initiation and progression of different types of human cancers. In this review, we discuss how m6A RNA methylation influences both the physiological and pathological progressions of hematopoietic, central nervous and reproductive systems. We will mainly focus on recent progress in identifying the biological functions and the underlying molecular mechanisms of m6A RNA methylation, its regulators and downstream target genes, during cancer progression in above systems. We propose that m6A RNA methylation process offer potential targets for cancer therapy in the future.

## Introduction

Epigenetics is a discipline that modulates heritable gene expression without DNA sequence changes. There are many types of well documented epigenetic modifications, such as DNA methylation, histone modification, chromatin remodeling, and non-coding RNA regulation, among which, the methylation modification of DNA and RNA is extremely important^1^. With the rapid development of specific antibodies and high-throughput sequencing, N6-methyladenine modification has made tremendous breakthroughs from prokaryotic bacteria to eukaryotic human being^2^. m6A modification has been identified as one of the post-transcriptional regulatory markers in different types of RNAs, such as messenger RNAs (mRNAs), transfer RNAs (tRNAs), ribosomal RNAs (rRNAs), circular RNAs (circRNAs), micro RNAs (miRNA), and long non-coding RNAs (lncRNAs)^3,4^. Furthermore, RNA m6A modification has been documented to play important roles for regulating RNA splicing, translation, stability, translocation, and the high-level structure^4–6^. The landscape of m6A in the transcriptome was first uncovered by next-generation sequencing (NGS)^7,8^, which showed that an average of 3–5 m6A modifications in each mRNA out of one-third of mammalian total mRNAs. Until now, m6A modification has been identified on almost every type of RNAs. MeRIP-seq (methylated RNA immunoprecipitation and sequencing) has been broadly used to profile m6A, detecting m6A containing region around ~100 nucleotide (nt) length^7,8^. However, the exact location of individual m6A site could not be efficiently and accurately identified, although multiple improved methods have been developed, such as PA-m6A-seq, miCLIP, and m6A-CLIP^9–12^. Most m6A sites were found in conserved motif DRACH (D = G/A/U, R = G/A, H = A/U/C)^13^, which have been frequently identified around the stop codon by whole-transcriptome m6A maps, indicating the potential functional roles for m6A^7,8,14^. To detect individual m6A sites, methyl-sensitive ligase, reverse transcriptase and selective dTTP (deoxythymidine triphosphate) analog have been applied^15–18^.

To examine the chemical properties of m6A sites, all of the above methods dependent on m6A-specific antibodies, which can also recognize the structurally similar cap modifications, leading to poor reproducibility and even controversial result. To solve this problem, Luo’s group recently has developed m6A-sensitive RNA-endoribonuclease–facilitated sequencing method or m6A–REFseq, which can identify transcriptomic m6A sites at specific motifs in single-base resolution^19^. By applying m6A-REF-seq to different tissues from human, mouse and rat, m6A modification was shown to be highly conserved at both gene and individual base level^19^. Similarly, Schwartz’s group has developed MAZTER-seq to quantify m6A stoichiometry at individual sites, which highly relies on the ability of the bacterial RNase MazF to cleave RNA immediately upstream of an “ACA” sequence, but not upstream of “m6A-CA”^20^. Then, Meyer’s group presented DART-Seq (deamination adjacent to RNA modification targets), also an antibody-independent method for detecting individual m6A sites. Meyer et al. altered the sequence near methylation sites by fusing APOBEC1 to the m6A-binding YTH domain, and detected subsequent editing events with RNA-Seq^21^. With the help of these methods based on single-base resolution, we will be able to apply precious primary patient material or limited primary cancer stem cells to examine the m6A profiling under different physiological and pathological conditions, which could be used as novel biomarkers in the future.

m6A is installed by methyltransferase complex including METTL3^22^, METTL14^23^, WTAP^24^, KIAA1429^25^, METTL16^26^, RBM15^27^, and ZC3H13^28^. m6A is removed by demethylases such as FTO^29^ and ALKBH5^30^. The m6A reader proteins can recognize the m6A-modified RNAs, which are divided into different protein families. One class of direct m6A readers proteins contain the YT521-B homology (YTH) domain^4^, and several heterogeneous nuclear ribonucleoproteins (HNRNPs) fall into the other category, which mainly regulate alternative splicing or processing of target transcripts^31^. IGF2 mRNA binding proteins (IGF2BP1/2/3) families^32^, and eukaryotic initiation factor (eIF) 3^33^, belong to another subfamily members. Different species choose different m6A reader proteins to carry out specific biological functions. For example, in order to adapt to a hypoxia environment, mammals living in high altitude select the YTHDF1, instead of YTHDF2/3, to resist hypoxia-induced cellular apoptosis in a Keap1-Nrf2 axis dependent manner^34^, suggesting that readers of individual m6A-modified RNAs can have both redundant and specific roles depending on different cellular context.

Numerous studies focusing on m6A RNA methylation have demonstrated that the regulators of m6A RNA methylation are involved in various human diseases, including Nonalcoholic fatty liver disease^35^, Azoospermia^36^, heart failure^37^, especially in human cancers^38^. In this review, we discuss how m6A RNA methylation influences both the physiological and pathological progressions of hematopoietic, central nervous and reproductive systems. We will mainly focus on recent progress in identifying the biological functions and the underlying molecular mechanisms of m6A RNA methylation, its regulators and downstream target genes, during cancer progression in above systems. We propose that m6A RNA methylation represents the potential target for cancer therapy in the future.

## The writers, erasers, and readers of m6A

m6A is installed by the m6A methyltransferases complex, which is a dynamical and reversible biological process (Table 1). As the first discovered methyltransferase and the core methyltransferase subunit, METTL3 plays a major catalytic role in m6A addition process. It has been well documented that METTL3 methylates its specific target transcripts and participates in various physiological processes, such as embryonic development^39^, brain development^40^, spermatogenesis^41^, cell reprogramming^39^, and T cell homeostasis^42^. METTL14, another key enzyme of methyltransferase complex, interacts with METTL3 and forms a stable heterodimer playing an essential role during m6A deposition on nuclear RNAs with increased catalytic efficacy^23,43^. In addition, deletion of METTL14 inhibits self-renewal and differentiation abilities of embryonic stem cell^44^. METTL16 has also been indicated to play as a methyltransferase for the U6 spliceosomal small nuclear RNA, which is required for SAM homeostasis^45^. Wilms’ tumor 1-associating protein (WTAP) forms protein complex with METTL3 and METTL14, deletion of which results in reduced RNA-binding capability of methyltransferase complex and embryonic differentiation^46^. It has been shown that the loss of RNA-binding motif protein 15 and its paralogue RBM15B (RBM15/RBM15B), results in impaired XIST-mediated gene silencing on the X chromosome during mammalian female development^47,48^. A recent study shows that KIAA1429 is able to recruit and guide the catalytic core methyltransferase components (METTL3/METTL14/WTAP) to specific RNA region for m6A methylation^49^. In *Drosophila* and mice, it has recently been documented that Zc3h13 (zinc finger CCCH domain-containing protein 13) interacts with Flacc (Fl(2)d-associated complex component) as a novel cofactor of m6A methyltransferase complex, which controls the overall m6A levels in sex determination in *Drosophila*^50^.

**Table 1:** The functional roles of m6A regulators in RNA metabolism.

Type	M6ARegulator	Function	References
m6A writer	METTL3	Catalyzes m6A modification	^22^
	METTL14	Assists METTL3 to recognize the subtract	^25^
	METTL16	Catalyzes m6A modification	^26^
	WTAP	Promotes METTL3-METTL14 heterodimer to the nuclear speckle	^24^
	KIAA1429	Guides the methyltransferase components to specific RNA region	^25^
	RBM15	Binds the m6A complex and recruit it to special RNA site	^47^
	ZC3H13	Bridges WTAP to the mRNA-binding factor Nito	^50^
m6A eraser	FTO	Removes m6A modification	^29^
	ALKBH5	Removes m6A modification	^30^
m6A reader	YTHDC1	Promotes RNA splicing and translocation	^55^
	HNRNPA2B1	Promotes primary microRNA processing	^60^
	HNRNPC	Mediates mRNA splicing	^58^
	YTHDF1	Promotes mRNA translation	^58^
	YTHDF2	Reduces mRNA stability	^232^
	YTHDF3	Mediates the translation or degradation	^232^
	YTHDC2	Enhances the translation of target RNA	^119^
	IGF2BP1/2/3	Enhances mRNA stability	^32^

The demethylases play as erasers to remove the m6A modifications in RNA. In 2011, one study reported that fat mass and obesity-associated protein (FTO) localizes in nuclear speckles to eliminate m6A residues in RNA^29^. A genome-wide screen for type 2 diabetes susceptibility genes identified a common variant in FTO predisposing to obesity through an effect on body mass index (BMI)^51^. FTO has also been reported to regulate the adipogenesis via regulating alternative splicing of adipogenic transcription factor RUNX1T1 (Runt-related transcription factor 1) in an m6A depend manner^52^. ALKBH5 was the second identified m6A demethylase, which has been shown to modulate mRNA export and RNA metabolism by reducing the m6A level in nuclear speckles^30^. Inactivation of ALKBH5 leads to male infertility in mice through appropriate m6A clearance in the nuclei of spermatocytes essential for correct splicing and the production of longer 3′-UTR mRNAs, and failure of which leads to aberrant splicing and accumulation of shorter transcripts^53^. DDX46, one member of DEAD-box (DDX) helicases, has been shown to inhibit antiviral innate responses by recruiting ALKBH5 and entrapping selected antiviral transcripts in the nucleus through erasing their m6A modification^54^.

**Fig. 1:**
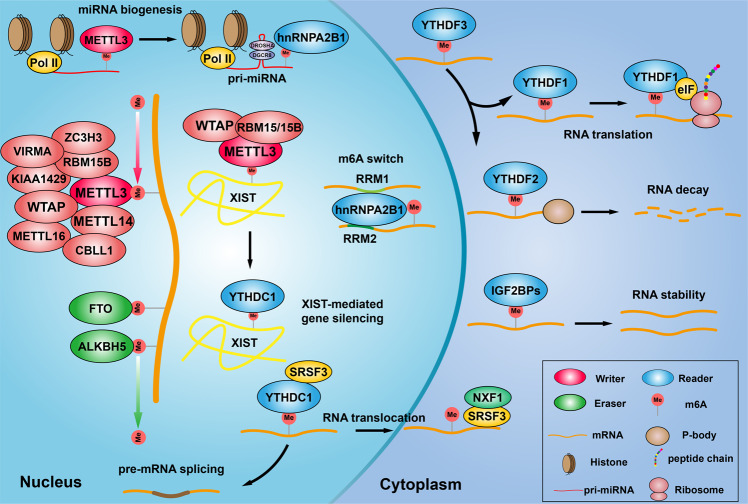
Introduction of m6A RNA modification complex. m6A methylation is catalyzed by the writer complex including METTL3, METTL14, METTL16, WTAP, VIRMA, RBM15/15B, CBLL1, KIAA1429, and ZC3H13. The m6A modification is erased by demethylases including FTO and ALKBH5. The m6A-modified RNA reader proteins include YTHDF1/2/3, YTHDC1/2, IGF2BP1/2/3, and HNRNPC/A2B1. m6A modification modulates miRNA biogenesis, XIST-dependent X chromosome inactivation, m6A switch, RNA translocation, pre-mRNA splicing, RNA translation, RNA decay and RNA stability

The m6A reader protein can recognize and bind to the m6A-modified transcript regulating gene expression through regulating diverse processes, such as mRNA stability^52^, mRNA splicing^55^, mRNA structure^56^, mRNA export^57^, translation efficiency^58^, and miRNA biogenesis^59^. Different readers have different m6A positioning function, nuclear m6A readers including YTHDC1, HNRNPA2B1, HNRNPC11, and HNRNPG. A recent study showed that YTHDC1 promotes exon inclusion in targeted transcripts by selectively recruiting pre-mRNA splicing factor SRSF3 while blocking SRSF10 mRNA binding^55^. The nuclear export of m6A methylated transcripts may be facilitated by YTHDC1 through interacting with nuclear transport receptors^57^. Another study demonstrated that YTHDC1 recognizes m6A-modified XIST to promote XIST-mediated X chromosome silencing^47^. It has been shown that hnRNP A2/B1 directly binds to and regulates the processing of m6A-modified transcripts, including subset of primary miRNA transcripts by interacting with the miRNA microprocessor complex protein DGCR8^31^. Similarly, a structure-based study revealed that hnRNP A2/B1 recognizes specific targets containing AGG and UAG motifs by RRM1 and RRM2 domains, respectively through an “m6A switch” mechanism^60^. Cytoplasmic m6A readers contain YTHDF1/2/3, YTHDC2, and IGF2BP1/2/3. YTHDF1 enhances translation by interacting with translation initiating factors and ribosomes^58^. YTHDF2 promotes targeted mRNA decay via recruiting RNA decay machinery factors^58^. YTHDF3 was reported to promote protein synthesis synergizing with YTHDF1, and regulate m6A-modified mRNA decay mediated by YTHDF2^61^. YTHDC2 plays an essential role on the switch from mitosis to meiosis during spermatogenesis^62^. IGF2BPs, the conserved single-stranded RNA-binding proteins (RBPs), structurally contain six canonical RNA-binding domains, two RNA recognition motif (RRM) domains, and four K homology (KH) domains^63^. IGF2BPs can enhance mRNA stability by binding to target transcripts through GG(m6A)C, a typical m6A motif^64^. Different reader proteins present different even opposite functions depending on different cellular context^65^ (Fig. 1).

## Hematopoietic, central nervous and reproductive systems

There are more than 10 different cell types with various functions in hematopoietic system. For examples, leukocytes are mainly involved in innate and acquired immunity; erythrocytes provide O_2_ and CO_2_ exchange and transport; megakaryocytes generate platelets during wound healing. Most importantly, hematopoietic or blood system, which sustains the nutrition and O_2_ bases for all the other animal tissues, develops in several waves during embryogenesis. The first wave emerges from the yolk sac at embryonic day 7.5 (E7.5) in mice^66,67^, which is characterized by the generation of nucleated megaloblastic erythroblasts referred to as primitive erythroblasts (PEs), together with diploid platelet progenitor cells and macrophages^68^. PEs are characterized by the predominant expression of embryonic globin genes, which eventually enucleate in the circulation^69,70^. The second wave has been linked to erythro-myeloid progenitors (EMPs) occurring in the yolk sac, which generates erythroid colonies similar to those derived from adult bone marrow (BM) and eventually migrate to the fetal liver and produce erythroblasts^66,71,72^. The third wave correlates with hematopoietic stem cells (HSCs) produced from the hemogenic endothelium identified in the dorsal aorta of the aorta-gonad-mesonephros (AGM) region, which can also emerge from umbilical, vitelline, yolk sac, cranial, and placental regions^67^. In addition, before HSCs finally become quiescent in the BM, they migrate to the fetal liver and undergo an expansion period^73^. Until now, many studies have demonstrated that epigenetic modification including DNA methylation, histone modification, and m6A modification plays important roles during hematopoietic development^74–84^.

The central nervous system (CNS) is a complex network composed of different types of nervous cells. During development, neural stem/progenitor cells have the potential to self-renewal, which can differentiate to produce various types of nervous cells, including neurons, astrocytes, and oligodendrocytes^85^. By using the retroviral lineage-tracing methods in mouse cortex, researchers found that neural stem/progenitor cells at midgestation generated small clones that could migrate quite widely^86–88^, and traced that these earlier infected clones could span multiple cortical layers with multipotency for neuronal production^89^. Embryonic neural progenitor cells (NPCs) are mainly classified into multipotent neural stem cells and transit amplifying/intermediate progenitor cells (IPCs)^90^. Hereafter, more results showed that most embryonic progenitors produce clones of neurons, while others of glia, but only a small population produce both neuronal and glial progeny^91–95^. Recently studies have demonstrated that neural stem/progenitor cells reside in the adult mammalian brain and contribute to brain plasticity throughout life^96,97^. These discoveries led to the concept that the CNS development is similar to that of the hematopoietic system, which relies on multipotent stem cells that have capacity for self-renewal and potency to produce more types of differentiated progeny. Yet today, although the key aspects of the mechanisms that underlie neural stem/progenitor cells remain enigmatic, many results have shown that various types of epigenetic modifications including m6A modification play critical roles in regulating the maintenance and differentiation of NSCs^98–106^.

Early in development, the germ cell lineage undergoes a series of complex developmental processes that culminate in the generation of the oocytesthe and spermatozoa^107^. In mouse, the germ cell lineage emerges in the most proximal posterior epiblasts at the beginning of gastrulation around embryonic day (E) 6.0^108,109^. Human PGCs migrate to the gonadal ridge (the precursor of the gonads) at approximately week 5 postfertilization and undergo proliferation before sexual differentiation^110,111^. The mouse primordial germ cells (PGCs) then migrate through the hindgut endoderm and mesentery and colonize the embryonic gonadal primordia^111–113^. In males, mPGCs continue to proliferate in embryonic testes until they enter into mitotic arrest and differentiate into gonocytes. In females, mPGCs undergo proliferation as cysts in embryonic ovaries until they enter into the first meiotic prophase and differentiate into oocytes^113–115^. Epigenetic reprogramming including m6A modification, DNA demethylation, and histone modification has been indicated to be crucial for this complex process^41,116–119^.

Based on the following facts that (1) the critical roles of hematopoietic, central nervous and reproductive systems for individual and the species, and (2) the continuous existence of multipotent stem cells under physiological conditions throughout life-time of animals and human, and (3) numerous documented findings corroborating that epigenetic reprogramming especially RNA m6A modification plays crucial roles in hematopoietic, central nervous and reproductive systems, we decided to mainly summarize and discuss how m6A RNA methylation influences both the physiological and pathological progressions in the above three systems (Table 2).

**Table 2:** The functional roles of m6A modification complex during normal development in hematopoietic, central nervous, and reproductive systems.

Organs	m6A regulator	Cells /Organisms	Effect of gene knockdown/depletion	Mechanism	References
Hematopoietic	METTL3	mHSPCs	↓Endothelial to hematopoietic transition	↓METTL3/YTHDF2↑/notch1a↓	^84^
	METTL3	HSPCs	↑Differentiation, ↓cell proliferation	↑METTL3/↑c-MYC/BCL2/ PTEN	^171^
	METTL3/L14	mESCs	↓Self-renewal	↓METTL3/14/HuR↓/↓IGFBP3	^43^
	YTHDF2	HSCs	↑Regeneration	↓YTHDF2/ ↑Wnt target genes	^124^
	METTL3	HSCs	↓Proliferation, ↓differentiation	↓METTL3/ ↑MDA5/RIG-I	^125^
Central nervous	METTL3/L14	RGCs	↑Neurogenesis, ↑cell cycle	↓METTL3/14/↑Tbr2/Neurog2/Neurod1	^106^
	METTL14	OPCs	↓Oligodendrocyte numbers	↓METTL14/↓NF155	^129^
	ALKBH5	RGCs	↑Proliferation, ↑differentiation	↓ALKBH5/ ↓Cacna2d3/Notch3/ Jam3	^131^
	FTO	NSCs	↓Proliferation, ↓differentiation	↓FTO/ ↓BDNF/PI3K/ Akt2/Akt3	^127^
	YTHDF1	Mice	↓Learning, memory defects	↓YTHDF1/↓Camk2a	^133^
	YTHDF1	Mouse	↓Axon guidance	↓YTHDF1/↓Robo3.1	^105^
	YTHDF2	mNSPC	↓Self-renewal	↓YTHDF1/↓JAK-STAT	^135^
	YTHDF1/3	Mouse	↓Synaptic transmission	↓YTHDF1/3/↓GluA1	^136^
	PRRC2A	OPCs	↓Proliferation	↓PRRC2A/↓Olig2	^137^
Reproductive	YTHDF2	Mouse	↓Oocyte maturation	↓YTHDF2/↓Trpc5	^139^
	METTL3	Zebrafish	↓Sperm motility	↓METTL14/↓11-KT/ 17β-E2	^140^
	YTHDC1	Germ cells	↓Oocyte growth, maturation	↓YTHDC1/↓CPSF6/ SRSF3	^141^
	ALKBH5	Mice	↓Fertility	↓ALKBH5/↑*Dnmt1*	^30^

## m6A RNA modification regulates hematopoietic development

**Fig. 2:**
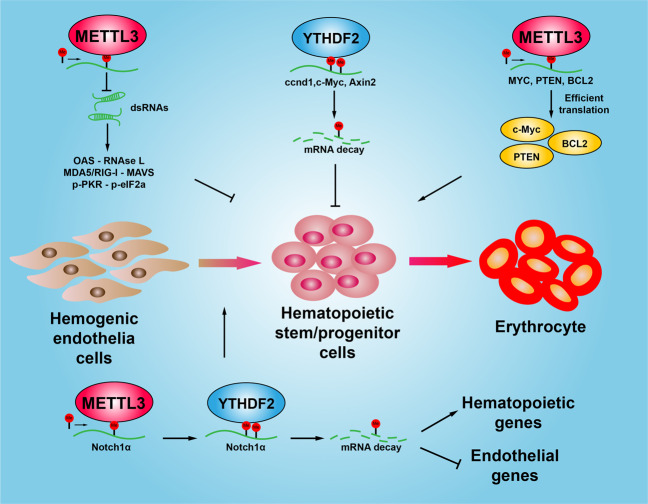
m6A RNA modification regulates hematopoietic system development. In the hematopoietic system, m6A methylation is essential for the proliferation and differentiation of hematopoietic stem/progenitor cells. Depletion of METTL3 promotes the formation of endogenous double-stranded RNAs (dsRNAs), which activates MDA5-RIG-I, PKR-eIF2α, and OAS-RNase L signaling pathways in hematopoietic stem/progenitor cells, resulting in hematopoietic development failure. YTHDF2 inhibits the Wnt signaling pathway by degrading the mRNA of ccnd1, c-Myc and Axin2, leading to reduced the proliferation and differentiation of hematopoietic stem/progenitor cells. METTL3 promotes the translation of c-Myc, PTEN, and BCL2 by increasing the methylation levels of reciprocal mRNAs, and promotes the proliferation of stem cells. METTL3 and YTHDF2 cooperate to inhibit the Notch signaling pathway in hematopoietic system

The hematopoietic system provides the lifelong supply of blood cells, which are derived from a rare population of multipotent HSCs^120^. Recent studies have indicated that m6A modification of RNAs plays pivotal roles during hematopoietic development, which is an essential process for blood system maturation^121^. The first evidence showed that m6A RNA modification determines cell fate during the endothelial-to-hematopoietic transition (EHT) to specify the earliest hematopoietic stem/progenitor cells (HSPCs) during *Zebrafish* embryogenesis, and METTL3 knockdown significantly inhibits EHT in a Notch signaling dependent manner^84^. Consistently, another study showed that depletion of METTL3 in vascular endothelial cells significantly represses the function of HSPCs^122^. However, shRNA-mediated knockdown of the METTL3 in human hematopoietic stem/progenitor cells (HSPCs) preferentially promotes cell differentiation but not cell proliferation, and vice versa^123^. Similarly, result has been shown that deletion of METTL3 and METTL14 leads to loss of self-renewal capability in mouse embryonic stem cells (mESCs)^43^. In addition, YTHDF2 was identified to regulate transcriptome switch during zebrafish maternal-to-zygotic transition^84^, and another study showed that YTHDF2 depletion is able to expand mouse and human hematopoietic stem cells dramatically, highlighting its potential role in clinical blood transplantation^124^. Furthermore, loss of METTL3 in the murine fetal liver can promote the formation of endogenous double-stranded RNAs (dsRNAs), which activates MDA5-RIG-I, PKR-eIF2α, and OAS-RNase L signaling pathways in hematopoietic stem/progenitor cells, resulting in hematopoietic failure and perinatal lethality^125^. The above findings indicate that m6A RNA methylation plays different roles during different developmental stages in various tissues. Thus, deciphering the underlying molecular mechanism will help us to better understand the mechanism of development and how to treat human diseases in the future (Fig. 2).

## m6A RNA methylation regulates CNS development

Emerging studies have indicated that m6A is more abundant in the CNS than that in other organs, which increases in overall abundance from the embryonic to adult brain, suggesting its critical roles during normal brain development and function^40^. Recent studies have demonstrated that m6A modification modulates adult neurogenesis in the mammalian midbrain^40^, embryonic brain development^126^, learning, and memory^127^. For example, depletion of either METTL3 or METTL14 in the embryonic mouse brains prolongs the cell cycle of radial glia cells and extends cortical neurogenesis into postnatal stages. In addition, m6A signaling has also been indicated to regulate human cortical neurogenesis in forebrain organoids^128^. In vivo conditional ablation of METTL14 leads to reduced oligodendrocyte numbers and CNS hypomyelination. Furthermore, depletion of Mettl14 disrupts postmitotic oligodendrocyte maturation^129^. Depleting METTL3 reduces memory consolidation ability in mouse hippocampus, while overexpression of METTL3 significantly enhances long-term memory consolidation^130^. Forced expression of METTL3 using lentivirus infection results in the disorganized structure of both Purkinje and glial cells.

**Fig. 3:**
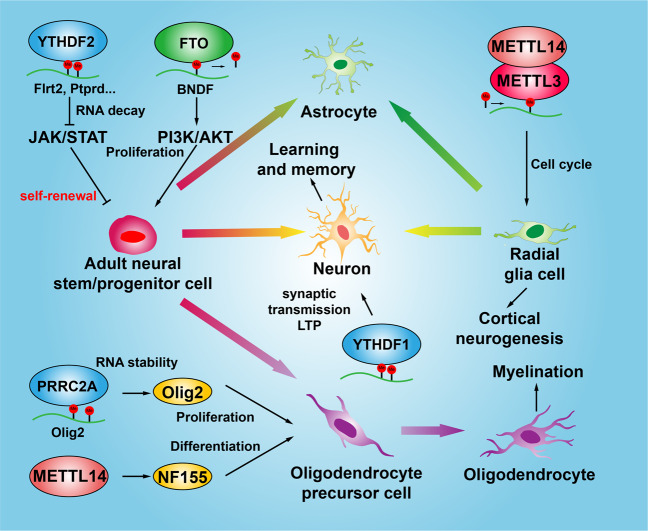
m6A RNA methylation regulates central nervous system development. Neural stem/progenitor cells have the potential to self-renewal, which can differentiate to produce various types of nervous cells, including neurons, astrocytes, and oligodendrocytes. YTHDF2 and FTO promote the self-renewal and proliferation of NSCs by regulating the JAK/STAT and PI3K/AKT signaling pathways. METTL14 and METTL3 promote cortical biogenesis by accelerating the cell cycle of radial glial cells. PRRC2A and METTL14 promote the proliferation and differentiation of oligodendrocyte precursor cells and the myelination process by promoting the expression of Olig2 and NF155, respectively. YTHDF1 regulates learning and memory by promoting synaptic transmission and transcription of LTP-related target genes in neurons

Under hypoxia conditions, deletion of ALKBH5 has been shown to promote cell proliferation and differentiation in the cerebellum by destroying the balance of RNA m6A methylation in different cell fate determination genes^131^. FTO was highly expressed in adult neural stem cells (NSCs) and neurons, loss of which not only reduces the proliferation and neuronal differentiation of NSCs, but also results in decreased brain size and body weight, leading to impaired learning and memory^127^. In the mouse brains, YTHDF1, one of the YTH domain-containing m6A-modified mRNA binding protein family members, whose mRNA is preferentially increased in the hippocampus, a key region in spatial learning and memory^132^. YTHDF1 was also found to promote protein translation of targeted m6A-modified transcripts in response to neuronal stimuli in the adult mouse hippocampus, thereby facilitating learning and memory^133^. Importantly, recent findings showed that YTHDF1 binds to and promotes translation of m6A-modified Robo3.1 transcript, which is essential for midline crossing of spinal commissural axons^105^. It has been shown that sciatic nerve lesion increases m6A-modified transcripts encoding many regeneration associated genes in the adult mouse dorsal root ganglion (DRG), and inhibition of METTL14 or YTHDF1 dramatically reduces injury induced functional axon regeneration^134^. Similar results showed that depletion of YTHDF2 inhibits NSCs self-renewal and spatiotemporal generation of neurons in embryonic neocortex, leading to lethality at late embryonic developmental stages^135^. One in vitro study showed that knockdown of YTHDF1 or YTHDF3 m6A readers, respectively, in cultured primary hippocampal neurons, causes decreased spine head volume and dampened spontaneous excitatory synaptic transmission^136^. A novel m6A reader PRRC2A (Proline rich coiled-coil 2A) was found to control oligodendrocyte specification and myelination. Conditional ablation of PRRC2A induces marked hypomyelination, cognitive defects in mouse, and decreased lifespan^137^ (Fig. 3).

## m6A RNA methylation regulates reproductive system development

A number of studies have shown that m6A modification plays pivotal roles in both oocyte maturation and spermatogenesis. Sexual reproduction starts with gametogenesis in both parents through meiosis, and fertilization then occurs with the merging of oocyte and sperm, which then initiates the developmental program of the offspring. Ivanova et al. reported that YTHDF2 is required for oocyte maturation and early zygotic development^138^. Qi et al. sequenced the N6-methyladenosine (m6A) modified mRNAs in various stages of oocytes in *Xenopus laevis*, and identified bunch of mRNAs with m6A peaks exhibiting lower protein levels than those of the hypomethylated mRNAs. The hypomethylated mRNAs were mainly involved in regulating the cell cycle and translation pathways, whereas the highly m6A-modified mRNAs were mainly associated with the protein phosphorylation, both of which are important for controlling oocyte meiotic maturation and early embryo development^139^. Using zebrafish as model, it has been demonstrated that METTL3 mutation disrupts gamete maturation and reduces fertility through decreasing overall m6A levels and expressions of critical genes required for sex hormone synthesis and gonadotropin signaling^140^. The nuclear m6A reader YTHDC1 has been indicated to interact with the pre-mRNA 3’ end processing factors CPSF6, SRSF3, and SRSF7, which regulates the processing of pre-mRNA transcripts in the oocyte nucleus during fetal development^141^.

**Fig. 4:**
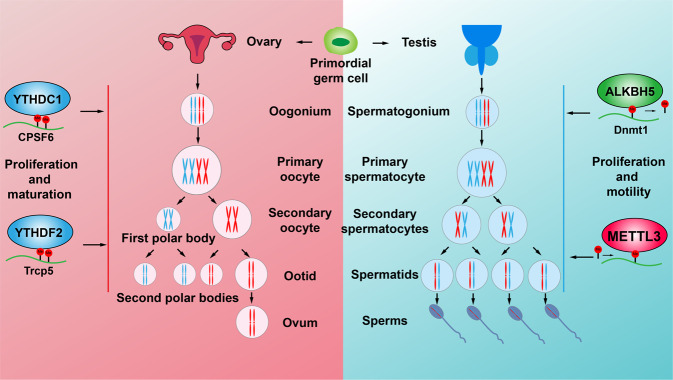
m6A RNA methylation regulates the reproductive system development. Primordial germ cells proliferate and differentiate into spermatocytes and oogonium in embryonic testes and ovaries, respectively. Spermatocytes and oogonium then undergo meiosis to become mature sperms and ovum, respectively. METTL3 and ALKBH5 regulate the levels of overall m6A mRNA methylation to promote the proliferation and motility of sperm cells. YTHDC1 and YTHDF1 regulate the maturation and translation of CPSF6 and Trcp5 transcripts, respectively, to promote the proliferation and maturation of oocytes

A recent study showed that m6A determines cell fate to specify the earliest hematopoietic stem cells during the endothelial-to-hematopoietic transition (EHT) in zebrafish, and YTHDF2 knockdown decelerates the decay of mA-modified maternal mRNAs and impedes zygotic genome activation [66]. In addition, depletion of YTHDF2 in mice is partially permissive, which results in the failure during oocyte maturation leading to female-specific infertility^138^. One study indicated that *Drosophila* Dmime4, the homolog of the Inducer of MEiosis 4 (IME4) gene catalyzing m6A modification in *Saccharomyces cerevisiae*, was highly expressed in ovaries and testes, which regulates the m6A installation of Notch transcript during follicle development^142^. The m6A eraser ALKBH5 has been identified to be highly expressed in male mice testes, ablation of which shows increased m6A in mRNAs mainly involved in p53 functional network leading to testicular atrophy, remarkably reduced rate of breeding and decreased fertility in Mice^30^. Consistently, a negative association between m6A modification and autophagy in Leydig cells (LCs) during testosterone synthesis was discovered, and a gradual decrease of METTL14 and an increase of ALKBH5 were detected in LCs during their differentiation by promoting translation of PPM1A but decreasing CAMKK2 RNA stability^36^. Furthermore, deficiency of the m6A reader protein YTHDC2 in mice has been shown to result in significant smaller testes and ovaries compared to control littermates^62^ (Fig. 4).

## m6A RNA methylation regulates other developmental processes

m6A methylation has been demonstrated to regulate various aspects of mRNA metabolism during adipose tissue expansion^143–145^. For examples, FTO has been demonstrated to associate with obesity and inhibit mitochondrial functions in adipocyte precursors via ARID5B-mediated repression of IRX3 (iroquois-related homeobox 3) and IRX5, and silencing FTO resulted in high m6A methylation on ATG5 and ATG7 mRNAs recognized by YTHDF2, leading to mRNA degradation and reduced autophagy and adipogenesis^146,147^. Consistently, FTO was uncovered to regulate exonic splicing of RUNX1T1 by regulating m6A modifications around splice sites, and the expression of cell-cycle regulators including CCNA2 and CDK2, and thereby modulates adipogenesis^52,148^. Furthermore, YTHDF1 was recently revealed to specifically recognize m6A-modified transcript of MTCH2 (mitochondrial carrier homology 2) in Jinhua pigs (obese-type breed with higher levels of intramuscular fat), which promotes MTCH2 mRNA translation and facilitates adipogenesis in intramuscular preadipocytes^149^. It has also been shown that fat-specific knockout of human antigen R (HuR), represses myogenesis program in brown fat by modulating the stability of Insig1, a negative regulator during adipogenesis^150^. Yao et al. showed that deletion of METTL3 in porcine bone marrow stem cells (BMSCs) activated JAK1/STAT5/C/EBPβ signaling pathway, and then promoted adipogenesis in an m6A-dependent manner^151^. Cen et al. also found that TRAF4 bound to PKM2 and negatively regulated mesenchymal stem cells (MSCs) adipogenesis by activating β-catenin signaling, while the reduced expression of TRAF4 during adipogenesis was controlled by ALKBH5^152^.

One study by Yuan’s group showed that deletion of METTL3 reduced the translational efficiency of MSCs lineage allocator Pth1r (parathyroid hormone receptor-1), and disrupted the PTH (parathyroid hormone)-induced osteogenic and adipogenic responses, leading to impaired bone formation^153^. Another study showed that METTL3 cooperates with ALKBH5 to regulate osteogenic differentiation, and knockdown of METTL3 reduces MYD88 expression, a critical upstream activator of NF-κB signaling, and therefore increases osteogenic progression^154^. In line with the above two findings, the inhibitory roles of METTL3 in osteogenesis by regulating RUNX2 or phosphatidylinositol 3-kinase/AKT (PI3K-Akt) signaling pathways, have also been documented by other groups^155,156^. In addition, the dynamic patterns of m6A methylation during liver development have been revealed by profiling transcriptome-wide m6A modification in porcine liver at three developmental stages: newborn (0 day), suckling (21 days) and adult (2 years)^157^.

## Pivotal roles of m6A RNA methylation in cancer

Besides the above findings that m6A RNA methylation is critical for normal hematopoietic, central nervous and reproductive systems development, aberrant m6A modification has also been indicated to be associated with various types of human cancers^65,158–164^. Here, we only focus on m6A RNA methylation in human cancers related to hematopoietic, central nervous, and reproductive systems (Table 3).

**Table 3:** The functional roles of RNA m6A modification complex in various types of human cancers

Cancer type	m6A regulator	Cell lines	Roles	Functions	Mechanism	References
AML	METTL3	CB-CD34 +	Oncogene	↑Proliferation, ↓differentiation	↑METTL3/↑c-MYC/BCL2	^123^
	METTL14	HPC, LSK	Oncogene	↑Proliferation, ↓differentiation	SPI-METTL14↑/↑MYB/MYC	^173^
	FTO	NK-AMLs	Oncogene	↑Proliferation	↑FTO/↑ASB2 / RARA	^174^
	FTO	NOMO-1, MA9.3ITD	Oncogene	↑Proliferation	↑FTO/↑MYC/CEBPA	^175^
	ALKBH5	MMC6	Oncogene	↑Self-renewal	↑ALKBH5 /↑TACC3	^177^
	ALKBH5	MOMO1	Oncogene	↑Self-renewal	↑KDM4C/↑ALKBH5↑/↑AXL	^178^
	YTHDF2	CNI, CNG	Oncogene	↓Apoptosis	↑YTHDF2/↑Tnfrsf2	^179^
	IGF1BP1	SKNO1, TANOUE	Oncogene	↓Differentiation, ↓ cell death	↑IGF2BP1 /↑HOXB4 / MYB	^180^
GBM	METTL3	PBT003, PBT707	Suppressor	↓Growth, ↓self-renewal	↑METTL3 /↑ADAM19	^184^
	METTL3	GSC17	Oncogene	↑Self-renewal, ↑proliferation	↑METTL3/↑SRSF↑/↑BCL-X/ NCOR2	^186^
	METTL3	U251, U87	Oncogene	↑Self-renewal, ↑DNA repair	↑METTL3/↑SOX2	^234^
	ALKBH5	GSC11, GSC23	Oncogene	↑Proliferation	↑FOXM1-AS-AKBH5/↑FOXM1	^185^
	hnRNPA1	U251, U87	Oncogene	↑Proliferation	↑hnRNPA1/↑Myc	^188^
	HnRNPA2	U251, A172	Oncogene	↑Proliferation, ↑migration,	↑HnRNPA2/↑p-STAT3 and MMP-2	^189^
	IGF2BP2	GSC88	Oncogene	↑Self-renewal, ↑proliferation	↑HIF1A-AS2-IGF2BP2/↑ HMGA1	^196^
	YTHDF2	GSC4121	Oncogene	↑Self-renewal,↑proliferation	↑YTHDF2/↑ MYC/IGFBP3	^257^
BC	METTL3	T24, EJ	Oncogene	↑Proliferation	↑METTL3/↑pri-miR221/222↓PTEN	^198^
	METTL3	BCa cells	Oncogene	↑Proliferation	↑METTL3/↑AFF4/NF-κB/MYC	^199^
	METTL3	T24,UMUC3	Oncogene	↑Proliferation, ↑metastasis	↑METTL3/YTHDF2/↑SETD7/KLF4	^200^
	METTL3	SW780, T24,	Oncogene	↑Proliferation,↑malignant t	↑METTL3/↑CDCP1	^201^
	METTL14	T24,UMUC3	Suppressor	↓Proliferation, ↓self-renewal,	↓METTL14/↑Notch1	^202^
OVC	ALKBH5	HEY, HO8910	Oncogene	↑Proliferation, ↓apoptosis	↑TLR4/↑ALKBH5/↑NANOG	^205^
	ALKBH5	SKOV3,COC1	Oncogene	↑Proliferation, ↓autophagy	↑ALKBH5/↑EGFR- AKT-mTOR	^204^
	YTHDF1	A2780, SKOV3	Oncogene	↑Proliferation, ↑migration,	↑YTHDF1/↑EIF3C	^38^
	HnRNPA2	A2780	Oncogene	↑Poliferation, ↑migration,	↑hnRNPA2B1/↑ Lin28B	^206^
	IGF2BP1	ES-2	Oncogene	↑Growth, ↑ invasion	↑SRF/IGF2BP1/↑ PDLIM7 / FOXK1	^207^
CVC	FTO	SiHa, c-33a	Oncogene	↑Chemo-radiotherapy resistance	↑FTO /↑β-catenin	^210^
PC	METTL3	PC-3	Oncogene	↑Proliferation, ↑migration,	↑METTL3/↑ LEF1	^211^

## m6A RNA methylation in acute myelocytic leukemia (AML)

AML is a malignant blood cell cancer of the immature myeloid hematopoietic cells in the bone marrow (BM), and a highly heterogeneous human disease with rising mortality^165^. More recently, numerous studies have focused on studying m6A RNA methylation in AM, as many factors were found to be involved in AML before uncovering their functional roles during m6A modification. For example, WTAP was initially identified as Wilms’ tumor gene (WT1) interactor, which is highly expressed in AML and associated with poor prognosis^166,167^. In line with its role in splicing regulation, WTAP was then demonstrated to form a complex with METTL3 and METTL14 proteins responsible for m6A modification^22,168^. Another example is RBM15, found to be highly expressed in hematopoietic system, its knockdown inhibited AML cell differentiation and induced cellular apoptosis, possibly through reducing Notch signaling^169,170^. Later on, Notch signaling pathway was uncovered as relevant m6A targets modified by RBM15 in AML^171^. Vu et al. provided evidence showing that METTL3 was highly expressed in AML, which elevates the installed m6A level in BCL2 and PTEN transcripts with, increased translation efficiency, leading to activation of AKT signaling to modulate the cell differentiation and self-renewal of acute myeloid leukemia cells^172^. In particular, METTL3 knockdown in MOLM-13 AML cell lines resulted in an m6A-dependent reduction of c-MYC, BCL2, and PTEN mRNA translation. c-MYC is a well-known oncogene in leukemia, whereas BCL2 and PTEN are negative regulators of PI3K/AKT pathway. Interestingly, forced expression of a non-functional METTL3 was also able to activate PI3K/AKT pathway, indicating that additional mechanism might be involved^123^.

**Fig. 5:**
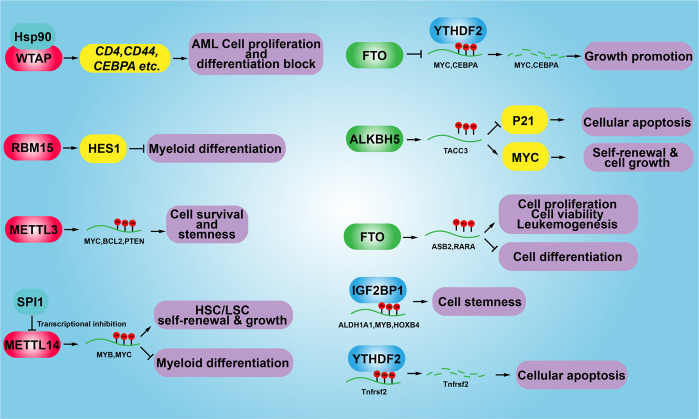
The functional role of m6A modification in human acute myelocytic leukemia (AML). During the development of AML, aberrant methylation or demethylation of the corresponding cancer-related genes contribute differentially during AML progression, including the cell proliferation, cell differentiation, cancer stem cell self-renewal and cellular apoptosis. HSC hematopoietic stem cells, LSC leukemia stem cells

Another recent finding showed that METTL14 plays an oncogenic role through elevating the expression of its mRNA targets including MYB and MYC in AML, while MEETL14 is negatively regulated by SPI1^173^. By contrast, with the m6A writers in AML, high-level expression of the eraser FTO demethylase and its oncogenic effect has also been demonstrated in AML. It was suggested that overexpression of FTO promotes cell proliferation and viability, while decreasing apoptosis and the global mRNA m6A level through reducing ASB2 and RARA expression^174^. Chen’s group found that R-2-hydroxyglutarate (R-2HG), inhibits FTO activity and increases global m6A RNA methylation levels in R-2HG-sensitive AML cells, which reduces the stability of MYC/CEBPA transcripts and related activities of relevant signaling pathways^175^. A study by Liu’s group showed that tyrosine kinase inhibitor (TKI) therapy resistant phenotypes depend on FTO overexpression and in turn m6A reduction in leukemia cells^176^. Interestingly, ALKBH5 was also found to be aberrantly overexpressed in AML, whose high expression correlates with poor prognosis in AML patients. A mechanistic study demonstrated that ALKBH5 is essential for the self-renewal of leukemia stem or initiating cells but not for normal hematopoietic, through post-transcriptional regulation of its target TACC3^177^. In addition, a recent study identified that KDM4C regulates ALKBH5 expression by increasing chromatin accessibility of ALKBH5 locus, and in turn promoting recruitment of MYB and Pol II. Furthermore, ALKBH5 regulates the stability of receptor tyrosine kinase AXL transcript in an m6A-dependent manner, indicating that ALKBH5 is involved in another signaling axis in AML^178^. Paris et al. found that the mRNA m6A reader YTHDF2 is overexpressed in AML, its decreased the clonogenic potential of AML cells and enhanced hematopoietic stem cells (HSC) activity by targeting to tumor necrosis factor receptor Tnfrsf2, which contributes to the overall integrity of LSC function^179^. Another mRNA m6A reader IGF2BP1 was found to be highly expressed in AML cells, and its decreased the proliferation and tumorigenic potential of leukemia cells through regulating the expression of the aldehyde dehydrogenase (ALDH1A1)^180^ (Fig. 5).

## m6A RNA methylation in brain tumor

Recently, increased evidence has demonstrated that writers, erasers, and readers of m6A RNA modification are associated with various types of human cancer, contributing to the self-renewal of cancer stem cell, promotion of cancer cell proliferation, and resistance to radio- or chemotherapy^181^. Gliomas are the common malignant primary brain tumors, and the standard therapy includes surgery followed by concurrent radiotherapy with temozolomide chemotherapy^182^. One bioinformatics study using The Chinese Glioma Genome Atlas (CGGA) and The Cancer Genome Atlas (TCGA) datasets showed that METTL3, METTL14, WTAP, RBM15, RBM15B, YTHDC2, YTHDF1, YTHDF2, YTHDF3, hnRNPA2B1, and hnRNPC are differentially expressed in gliomas accompanied with increased WHO grade, indicating that m6A RNA methylation regulators play important role in glioma malignancy^183^. For example, METTL3 or METTL14 knockdown robustly promotes glioma stem cell (GSC) self-renewal and tumorigenesis through increasing expression of stemness marker genes (e.g., ADAM19), while overexpression of METTL3 or inhibition of FTO inhibits GSC self-renewal and grafted tumor formation in vivo^184^. Consistently, m6A demethylase ALKBH5 has been identified to be highly expressed in GSC, the silencing of which suppressed the cell proliferation of patient-derived GSC by increasing FOXM1 expression resulted from reduced m6A methylation levels and increased HuR binding in FOXM1 transcript^185^. However, a controversial study showed that elevated expression of METTL3 is associated with the clinical aggressiveness of malignant gliomas, METTL3 knockdown or overexpression of dominant-negative mutant METTL3 suppressed the growth and self-renewal of GSC. A mechanistic study showed that METTL3 depletion decreased m6A modification levels of serine- and arginine-rich splicing factors (SRSF), leading to YTHDC1-dependent nonsense mediated mRNA decay (NMD) of SRSF transcripts^186^.

**Fig. 6:**
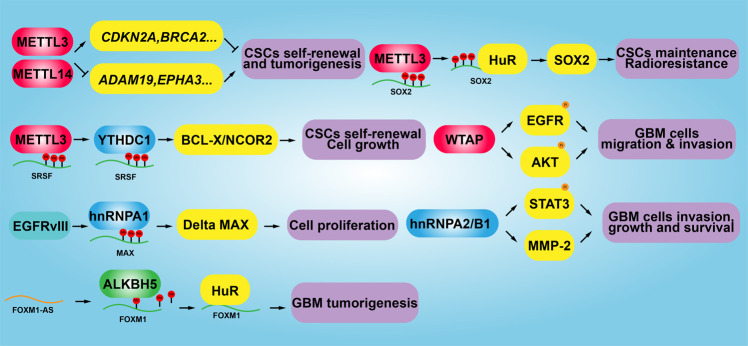
The functional role of m6A modification in gliomas. In gliomas, m6A modifiers regulate the cell proliferation, cell invasion, cell migration and cancers stem cell maintenance by targeting to multiple critical cancer-related genes. CSCs Cancer stem cells

WTAP, identified as a nuclear protein associating with the regulation of cell proliferation and apoptosis, was recently uncovered to be overexpressed in GBM, ablation of which reduces cancerous cell migration and invasion possibly by regulating the EGFR activity^187^. As a nuclear localized m6A reader protein, hnRNPA1 is upregulated by EGFRvIII, leading to increased glycolytic gene expression and shorter survival time in GBM. More evidence demonstrated that hnRNPA1 promotes splicing of Max transcript and then generating Delta Max, which enhances Myc-dependent cell transformation^188^. Similarly, hnRNPA2 was uncovered to be highly expressed in gliomas, which is associated with advanced glioma grades. Knockdown of hnRNPA2 can reduce cancerous cell viability, migration, invasion, and chemoresistance for TMZ, by reducing the expressions of phospho-STAT3 and MMP-2, which have been considered as oncogenic drivers in gliomas^189^. Another study showed that hnRNPA2 high expression causes PKM2 accumulation, suggesting that hnRNPA2 is required for cell proliferation and GBM progression^190^. Insulin-like growth factor 2 mRNA-binding protein 2 (IG2BP2) was recently found to support GSC and neural stem cell specification by binding to let-7 miRNA recognition elements (MREs) and preventing let-7 targeted gene silencing^191^. Consistent with this observation, another study demonstrated that IGF2BP2 binds to several mRNAs that encode mitochondrial respiratory chain complex subunits, such as complex I (NADH: ubiquinone oxidoreductase) factors, to regulate oxidative phosphorylation (OXPHOS), leading to increased self-renewal of GSC and tumor initiation by^192^. Forced expression of miR-873 in GBM cancerous cell lines dramatically reduces the cell proliferation, migration, and invasion by decreasing IGF2BP1 expression^193^. Furthermore, miRNA138 was identified to be downregulated in low grade gliomas (LGG), which is associated with worse clinical outcome. Ectopic expression of miR-138 suppresses cell proliferation, invasion, and xenograft tumor formation, by directly repressing IGF2BP2 expression^194^. A similar result found that miRNA129-1 acts as a tumor suppressor and induces cell-cycle arrest of GBM cells through targeting IGF2BP3 and MAPK1^195^. Except for miRNAs, long no-coding RNA HIF1A-AS2 was uncovered to be upregulated in mesenchymal GSC, whose deregulation affects GSC growth and self-renewal by interacting with IGF2BP2 to maintain the expression of HMGA1^196^. Circular RNA (circRNA) circHIPK3 was reported to be upregulated in gliomas, which is associated with poor prognosis. circHIPK3 promotes glioma cell proliferation and invasion via interacting with miR-654 leading to the stabilization of IGF2BP3^197^ (Fig. 6).

## m6A RNA methylation in reproductive system related cancers

In bladder cancer, METTL3 binds to DGCR8 and positively accelerates pri-miR221/222 maturation process, which results in the reduction of PTEN and ultimately increased cell proliferation^198^. Consistent findings demonstrated that METTL3 is significantly upregulated in bladder cancer, knockdown of which dramatically repressed cancerous cell proliferation, invasion, and xenograft tumor formation via AFF4/NF-kB/Myc axis signaling pathway^199^. Interestingly, a recent study showed that METTL3 recruits YTHDF2 to degrade the m6A-modified transcripts of the tumor suppressors including SETD7 and KLF4, contributing to the progression of bladder cancer^200^. Furthermore, new evidence indicated that METTL3 cooperates with YTHDF1 to promote the translation of oncogene CDCP1 in bladder cancer^201^. However, METTL14 has been identified to be decreased in bladder cancer and related tumor initiating cells (TICs), knockdown of which promotes the cell proliferation, self-renewal, metastasis and tumor initiating capacity through reducing the stability of m6A-modified Notch1 mRNA^202^.

**Fig. 7:**
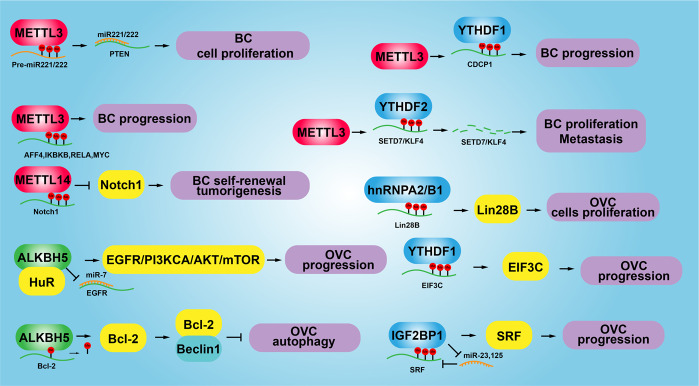
The functional role of m6A mRNA modification in reproductive system related cancers. Aberrant m6A methylation or demethylation of the corresponding cancer-related genes plays different roles in bladder cancer and ovarian cancer. The cell proliferation, cell metastasis, cancer stem cell maintenance, cellular apoptosis, and cell invasion were regulated by different m6A modifiers. BC bladder cancer, OVC ovarian cancer

Decreased mRNA and protein expressions of FTO results in an increase of the overall m6A levels in mRNA leading to premature ovarian insufficiency^203^. ALKBH5 is elevated in epithelial ovarian cancer, silencing of ALKBH5 enhances the autophagy signaling and inhibits the proliferation and invasion abilities of ovarian cancer cells by reducing EGFR/PI3K/AKT signaling activity through physically interacting with HuR^204^. Additional evidence showed that ALKBH5 was highly expressed in ovarian cancer tissue, but decreased in cancerous cell lines, whose expression pattern is consistent with Toll-like receptor (TLR4). However, when ovarian cancer cells were co-cultured with M2 macrophages, the expressions of ALKBH5 and TLR4 were both increased. TLR4 was then uncovered to increase ALKBH5 expression via activating NF-kB signaling pathway mediated by m6A modification^205^. Furthermore, YTHDF1 and hnRNPA2 promote ovarian cancer progression through elevating EIF3C and Lin 28B expression, respectively^38,206^. In addition, IGF2BP1 was reported to impair the miRNA-directed decay of the SRF mRNA and then promote the expression of SRF in a m6A-dependent manner, sustaining the expression of PDLIM7 and FOXK1, which promotes tumor cell growth and cell invasion of ovarian cancer^207^. IGF2BP3, as another RNA-binding protein modulating gene expression by post-transcriptional action, was found to be overexpressed in Ovarian Clear Cell Carcinoma (OCCC), which elevates the proliferation and tumorigenicity of OCCC^208^. The reduced m6A level has been identified in the cervical cancer comparing with the adjacent normal tissue, and increased FTO expression in cervical squamous cell carcinoma (CSCC) tissues can enhance the chemo-radiotherapy resistance of cancer cells by targeting β-catenin through mRNA demethylation^209,210^. While, METTL3 has been found to be upregulated in prostate cancer, and METTL3 increases the cell proliferation and migration of prostate cancer cells by activating expression of LEF1 or Myc in an m6A methylation dependent manner^211,212^ (Fig. 7).

## m6A RNA methylation in other human diseases

Considering the pivotal roles of m6A modification on gene expression, m6A has been increasingly implicated in other human diseases, including psychiatric disorders, metabolic syndrome, and cardiovascular diseases. For example, m6A modification was documented to be impaired in major depressive disorder (MDD) patients, and depletion of METTL3 or FTO in adult neurons has been shown to alter the m6A epitranscriptome and increase fear memory^213^. YTHDC2 has recently been identified as one of the autism spectrum disorder (ASD) risk gene by comparing ASD family with healthy control group obtained from East Asian populations^214^, and polymorphisms in ZC3H13 have also been shown to associate with schizophrenia^215^. Consistently, the overall m6A level was elevated in the cortex and the hippocampus of APP/PS1 (Alzheimer’s disease) mice compared to C57BL/6 control mice, and FTO was found to interact with APOE, which is associated with Alzheimer’s disease risk in a prospective cohort study^216,217^. In addition, circSTAG1 was recently identified to be decreased in the chronic unpredictable stress-treated mouse hippocampus and in peripheral blood of patients with major depressive disorder, and forced expression of circSTAG1 can attenuate astrocyte dysfunction and depressive-like behaviors in a ALKBH5/FAAH axis dependent manner^218^.

One study has identified FTO as one of the type 2 diabetes susceptibility genes regulating the adipogenesis^51,52^. Knockdown of Zinc finger protein 217 (ZFP217) was shown to increase expression of METTL3, which led to YTHDF2-mediated decay of cyclin D1 transcript and reduced mitotic clonal expansion and adipogenesis^219^. METTL3 was also found to be increased in the liver tissues from patients with type 2 diabetes (T2D), and METTL3 depletion decreased the m6A methylated mRNA of Fatty acid synthase (Fasn), leading to repressed fatty acid metabolism^220^. Consistently, it has been shown that METTL3 expression and overall mRNA m6A methylation level was upregulated in the livers of mice with high fat diet (HFD)-induced metabolic disorders, while hepatocyte-specific knockout of METTL3 alleviated lipid accumulation and improved insulin sensitivity^221^. Furthermore, YTHDC2 was shown to be significantly reduced in multiple obese mouse models and nonalcoholic fatty liver disease (NAFLD) patients, and knockdown of YTHDC2 led to excessive triglycerides (TGs) accumulation in hepatocytes, via inhibiting the expressions of lipogenic genes including sterol regulatory element-binding protein 1c, fatty acid synthase and acetyl-CoA carboxylase 1^222^. Importantly, m6A sequencing in human type 2 diabetes islets revealed multiple hypomethylated transcripts involved in insulin secretion, cell-cycle progression and the Insulin/IGF1 pathway, and METTL14 knockout in mouse β-cell resulted in reduced m6A levels, leading to similar islet phenotype in human T2D and mortality^223^.

There is emerging evidence showing that m6A modification is closely related to the occurrence and progression of cardiovascular diseases, such as cardiac hypertrophy, heart failure, ischemic heart disease and pulmonary hypertension^224,225^. For example, the level of m6A methylation was significantly increased on transcripts involved in regulating kinases and intracellular signaling pathways in cardiomyocyte upon hypertrophic stimulation, and overexpression of METTL3 has been shown to promote cardiomyocyte hypertrophy both in vitro and in vivo^226^. In line with this finding, FTO was uncovered to be decreased in failing mammalian hearts and hypoxic cardiomyocytes, and cardiomyocyte restricted knockout of FTO showed an impaired cardiac function in mice, via selectively demethylating and enhancing the stability of Serca2a mRNA^37,227^. One more study showed that m6A modification level was increased in hypoxia/reoxygenation (H/R) treated cardiomyocytes and ischemia/reperfusion (I/R)-treated mice heart, and deletion of METTL3 enhanced autophagic flux and inhibited apoptosis in H/R-treated cardiomyocytes dependent on TFEB, a master regulator of lysosomal biogenesis and autophagy^228^. Furthermore, METTL14 expression was indentified to be upregulated in calcific arteries and primary human artery smooth muscle cell (HASMC) induced by indoxyl sulfate, which selectively methylates vascular osteogenic transcripts^229^. In addition, total Panax notoginseng saponin (TPNS) was recently found to inhibit the intimal hyperplasia and reverse the reduced m6A quantity in balloon catheter-injured rat carotid artery, by increasing WTAP expression, indicating that WTAP may serve as a novel biomarker and therapeutic target for arterial stenosis in the future^230^.

## Conclusion and future direction

Although numerous findings related to the functional roles of RNA m6A modification have been reported, there are many major knowledge gaps remained to be filled. Whether the nucleic acid position (e.g., 5ʹ-UTR, coding sequence, 3′-UTR, splicing sites) and the level of m6A RNA methylation in RNA transcripts affect the recognition and functions of different m6A reader proteins remains elusive. Various m6A readers exhibit opposite functions. For example, YTHDF2 can promote the degradation of m6A methylated transcripts, whereas IGF2BP proteins can protect their targeted m6A-modified mRNAs from degradation under physiological and pathological conditions^64^. The competition and cooperation relationship among different reader proteins should be further clarified. Regarding YTH containing family members, YTHDF1 interacts with YTHDF3 in an m6A-independent manner to promote targeted mRNAs, while YTHDF3 interacts with YTHDF2 to promote the degradation of targeted transcripts^231,232^. A unified functional model for YTHDFs has recently been described^233^. YTHDF proteins can bind the same m6A-modified mRNAs rather than different mRNAs, and YTHDFs have been found not to be able to induce translation in HeLa cells. Interestingly, in this working model, different YTHDF proteins act redundantly to mediate mRNA degradation and cellular differentiation^233^. Elevated levels of m6A RNA methylation can promote the proliferation of some types of cancer cells. However, overexpression of METTL3 and METTL14 has also been indicated to suppress cancer cell growth^184,185,234^. Even in the same type of human cancer, different groups have drawn controversial conclusions, which might be cellular context dependent or differential expression levels of interested genes^184,185,234^. In addition, recent finding by our group found that, under normoxic conditions, YTHDF1 is highly expressed in non-small cell lung cancer cancerous tissues and cell lines to promote cell proliferation via increasing cell-cycle related factor expression. However, under hypoxic conditions or chemotherapy stressful conditions, YTHDF1 is downregulated which leads to reduced Keap1 mRNA translational efficiency and Nrf2 protein stabilization^34^.

Recently, Somasundaram’s group found that METTL3-mediated m6A modification in Sox2 is crucial for glioma stem-like cells (GSCs) maintenance and dedifferentiation both in vitro and in vivo^234^. In line with this finding, other studies also showed an oncogenic role for METTL3^142,235–242^. However, multiple reports have demonstrated that METTL3 and the associated m6A modification inhibited tumorigenicity of GSCs, while high levels of ALKBH5 was critical for promoting tumorigenicity of GSCs^184,185^. The phenotypic differences observed could be argued by different reliance on m6A-modified RNAs in different cell state and the dominant RNA species in GBMs. Furthermore, Somasundaram’s group performed a comparison of expression levels of METTL3/METTL14 and FTO/ALKBH5 in multiple GBM transcriptome and pan caner datasets, and found that METTL3 is significantly upregulated in the majority of tumors compared with METTL14, FTO, and ALKBH5. Based on the fact that readers mainly defined the fate of m6A-modified mRNAs, their cell-specific expression levels and mRNA binding affinity in GBM will eventually determine the differential functions of m6A modification. In addition, controversial findings about the role of m6A modification were also detected in mouse embryonic development, which could be explained by using naïve ESCs and primed epiblast stem cells (EpiSC) for functional assays^39,43,243,244^. m6A modification preserves the stability of EpiSC, while promotes naïve ESCs to undergo epiblast transition by reducing expressions of stem marker genes^245^. Therefore, further experiments are required for explaining the above discrepancies by performing context and compartment dependent functional studies of m6A modification. The antibody-independent methods for detecting individual m6A sites will also benefit our understanding of these conflict findings resulted from the inter-play between m6A modification and reciprocal reader proteins. In addition, numerous studies have been focused on the functional roles of RNA m6A modifiers in AML but not Chronic myeloid leukemia (CML), resulted from the formation of the BCR-ABL1 fusion protein^246^. The cross-talk between RNA m6A modification and BCR-ABL1 should be further characterized in future.

Considering different cancers with different genetic background, m6A RNA methylation can regulate oncogene expression (e.g., RNA processing, splicing, translocation, degradation), cancer stem/initiating cell pluripotency, cell differentiation, cell proliferation, migration, angiogenesis, and tumor microenvironment to control cancer progression. Therefore, targeting m6A RNA modification factors could provide potential therapeutic target for various human cancers. For example, R-2-hydroxyglutarate (R-2HG), produced by mutant isocitrate dehydrogenase 1/2 enzymes, was recently found to be able to restrain leukemia cell proliferation and induced cell apoptosis by targeting FTO/m6A/MYC/CEBPA signaling^247^. A non-steroidal anti-inflammatory drug, meclofenamic acid (MA) and N-(5-Chloro-2,4-dihydroxyphenyl)-1-phenylcyclobutanecarboxamide have recently been identified as selective inhibitors for FTO by competing with FTO binding for the m6A containing nucleic acid^248,249^. Two promising FTO inhibitors named as FB23 and FB23-2 were developed to selectively inhibit FTO’s m6A demethylase activity, which dramatically suppresses the proliferation and promotes apoptosis of AML cells^250^. Furthermore, two compounds referred as CS1 and CS2 were shown to bind tightly to FTO protein and block its catalytic pocket, thereby exhibited strong antitumor effects in multiple types of cancers^251^. MO-I-500 was recently identified as a selective inhibitor of FTO, which was shown to repress the proliferation of triple-negative breast cancer cells^252^. A more recent study validated the therapeutic potential of targeted mRNA demethylation using an engineered dCas13b-ALKBH5 fusion protein^253^.

Using lymphopaenic mouse adoptive transfer model, m6A mRNA methylation has been identified to control T cell homeostasis by targeting the IL-7/STAT5/SOCS signaling pathways^42^. Interestingly, METTL3 has also been reported to promote dendritic cell (DC) activation and function through upregulating the expression of costimulatory molecules CD40, CD80, and cytokine IL-12^254^. Furthermore, a study by He’s group recently showed that durable neoantigen-specific immunity is regulated by mRNA m6A modification in a YTHDF1 dependent manner, and *Ythdf1*-depletion mice exhibited an elevated antigen-specific CD8+ T cell antitumor response, and an increased therapeutic efficacy of PD-L1 checkpoint blockade^255^. In addition, FTO level was found to be increased in human melanoma, knockdown of which increases m6A methylation in PD-1, CXCR4, and SOX10, leading to increased RNA decay through the m6A reader YTHDF2. Therefore, FTO depletion sensitizes melanoma cells to interferon gamma (IFNγ) and anti-PD-1 treatment in mice^256^. These data suggest that targeting m6A mRNA methylation key regulators could promote anti-cancer therapies in the future.

## References

[1] Jones PA, Issa J-P, Baylin S (2016). Targeting the cancer epigenome for therapy. Nat. Rev. Genet..

[2] Liu H (2019). Accurate detection of mA RNA modifications in native RNA sequences. Nat. Commun..

[3] Liu N, Pan T (2016). N6-methyladenosine-encoded epitranscriptomics. Nat. Struct. Mol. Biol..

[4] Shi H, Wei J, He C (2019). Where, when, and how: context-dependent functions of rna methylation writers, readers, and erasers. Mol. cell..

[5] Yang Y, Hsu PJ, Chen YS, Yang YG (2018). Dynamic transcriptomic m(6)A decoration: writers, erasers, readers and functions in RNA metabolism. Cell Res..

[6] Zhao BS, Roundtree IA, He C (2017). Post-transcriptional gene regulation by mRNA modifications. *Nature reviews*. Mol. cell Biol..

[7] Dominissini D (2012). Topology of the human and mouse m6A RNA methylomes revealed by m6A-seq. Nature.

[8] Meyer KD (2012). Comprehensive analysis of mRNA methylation reveals enrichment in 3’ UTRs and near stop codons. Cell.

[9] Chen K (2015). High-resolution N(6) -methyladenosine (m(6) A) map using photo-crosslinking-assisted m(6) A sequencing. Angew. Chem. Int Ed. Engl..

[10] Linder B (2015). Single-nucleotide-resolution mapping of m6A and m6Am throughout the transcriptome. Nat. Methods.

[11] Ke S (2015). A majority of m6A residues are in the last exons, allowing the potential for 3’ UTR regulation. Genes Dev..

[12] Li X, Xiong X, Yi C (2016). Epitranscriptome sequencing technologies: decoding RNA modifications. Nat. Methods.

[13] Fu Y, Dominissini D, Rechavi G, He C (2014). Gene expression regulation mediated through reversible m^6^A RNA methylation. Nat. Rev. Genet..

[14] Schwartz S (2013). High-resolution mapping reveals a conserved, widespread, dynamic mRNA methylation program in yeast meiosis. Cell.

[15] Harcourt EM (2013). Identification of a selective polymerase enables detection of N(6)-methyladenosine in RNA. J. Am. Chem. Soc..

[16] Wang S (2016). N(6)-Methyladenine hinders RNA- and DNA-directed DNA synthesis: application in human rRNA methylation analysis of clinical specimens. Chem. Sci..

[17] Shu X (2017). N(6)-allyladenosine: a new small molecule for RNA labeling identified by mutation assay. J. Am. Chem. Soc..

[18] Hong T (2018). Precise antibody-independent m6A identification via 4SedTTP-involved and FTO-assisted strategy at single-nucleotide resolution. J. Am. Chem. Soc..

[19] Zhang Z (2019). Single-base mapping of m(6)A by an antibody-independent method. Sci. Adv..

[20] Garcia-Campos MA (2019). Deciphering the “m(6)A Code” via antibody-independent quantitative profiling. Cell.

[21] Meyer KD (2019). DART-seq: an antibody-free method for global m(6)A detection. Nat. Methods.

[22] Bokar JA (1997). Purification and cDNA cloning of the AdoMet-binding subunit of the human mRNA (N6-adenosine)-methyltransferase. RNA.

[23] Liu J (2014). A METTL3-METTL14 complex mediates mammalian nuclear RNA N6-adenosine methylation. Nat. Chem. Biol..

[24] Agarwala SD, Blitzblau HG, Hochwagen A, Fink GR (2012). RNA methylation by the MIS complex regulates a cell fate decision in yeast. PLoS Genet..

[25] Schwartz S (2014). Perturbation of m6A writers reveals two distinct classes of mRNA methylation at internal and 5’ sites. Cell Rep..

[26] Pendleton KE (2017). The U6 snRNA m(6)A Methyltransferase METTL16 Regulates SAM Synthetase Intron Retention. Cell.

[27] Patil DP (2016). m 6 A RNA methylation promotes XIST-mediated transcriptional repression. Nature.

[28] Wen J (2018). Zc3h13 regulates nuclear rna ma methylation and mouse embryonic stem cell self-renewal. Mol. cell..

[29] Jia G (2011). N6-methyladenosine in nuclear RNA is a major substrate of the obesity-associated FTO. Nat. Chem. Biol..

[30] Zheng G (2013). ALKBH5 is a mammalian RNA demethylase that impacts RNA metabolism and mouse fertility. Mol. Cell..

[31] Alarcón CR (2015). HNRNPA2B1 Is a mediator of m(6)A-dependent nuclear RNA processing events. Cell.

[32] Huang H (2018). Recognition of RNA N(6)-methyladenosine by IGF2BP proteins enhances mRNA stability and translation. Nat. Cell Biol..

[33] Du H (2016). YTHDF2 destabilizes m(6)A-containing RNA through direct recruitment of the CCR4-NOT deadenylase complex. Nat. Commun..

[34] Shi Y (2019). YTHDF1 links hypoxia adaptation and non-small cell lung cancer progression. Nat. Commun..

[35] Zhou, B. et al. N -methyladenosine reader protein Ythdc2 suppresses liver steatosis via regulation of mRNA stability of lipogenic genes. *Hepatology (Baltimore, Md.)* (2020). 10.1002/hep.31220 32150756

[36] Chen, Y. et al. mA mRNA methylation regulates testosterone synthesis through modulating autophagy in Leydig cells. *Autophagy* 1–19, 10.1080/15548627.2020.1720431 (2020). 10.1080/15548627.2020.1720431 PMC8007139 31983283

[37] Berulava T (2020). Changes in m6A RNA methylation contribute to heart failure progression by modulating translation. Eur. J. Heart Fail..

[38] Liu T (2020). The m6A reader YTHDF1 promotes ovarian cancer progression via augmenting EIF3C translation. Nucleic Acids Res..

[39] Aguilo F (2015). Coordination of m(6)A mRNA methylation and gene transcription by ZFP217 regulates pluripotency and reprogramming. Cell. Stem Cell..

[40] Hess ME (2013). The fat mass and obesity associated gene (Fto) regulates activity of the dopaminergic midbrain circuitry. Nat. Neurosci..

[41] Xu K (2017). Mettl3-mediated m(6)A regulates spermatogonial differentiation and meiosis initiation. Cell Res..

[42] Li HB (2017). m(6)A mRNA methylation controls T cell homeostasis by targeting the IL-7/STAT5/SOCS pathways. Nature.

[43] Wang Y (2014). N6-methyladenosine modification destabilizes developmental regulators in embryonic stem cells. Nat. Cell Biol..

[44] Geula S (2015). Stem cells. m6A mRNA methylation facilitates resolution of naïve pluripotency toward differentiation. Science.

[45] Pendleton KE (2017). The U6 snRNA mA methyltransferase METTL16 regulates SAM synthetase intron retention. Cell.

[46] Ping X-L (2014). Mammalian WTAP is a regulatory subunit of the RNA N6-methyladenosine methyltransferase. Cell Res..

[47] Patil DP (2016). m(6)A RNA methylation promotes XIST-mediated transcriptional repression. Nature.

[48] Penny GD (1996). Requirement for Xist in X chromosome inactivation. Nature.

[49] Yue Y (2018). VIRMA mediates preferential mA mRNA methylation in 3’UTR and near stop codon and associates with alternative polyadenylation. Cell Discov..

[50] Wen J (2018). Zc3h13 regulates nuclear RNA m(6)A methylation and mouse embryonic stem cell self-renewal. Mol. Cell..

[51] Frayling TM (2007). A common variant in the FTO gene is associated with body mass index and predisposes to childhood and adult obesity. Science.

[52] Zhao X (2014). FTO-dependent demethylation of N6-methyladenosine regulates mRNA splicing and is required for adipogenesis. Cell Res..

[53] Tang C (2018). ALKBH5-dependent m6A demethylation controls splicing and stability of long 3’-UTR mRNAs in male germ cells. Proc. Natl Acad. Sci. USA.

[54] Zheng Q (2017). The RNA helicase DDX46 inhibits innate immunity by entrapping mA-demethylated antiviral transcripts in the nucleus. Nat. Immunol..

[55] Xiao W (2016). Nuclear m(6)A reader YTHDC1 regulates mRNA splicing. Mol. Cell..

[56] Spitale RC (2015). Structural imprints in vivo decode RNA regulatory mechanisms. Nature.

[57] Roundtree, I. A. et al. YTHDC1 mediates nuclear export of N6-methyladenosine methylated mRNAs. **6**, e31311 (2017). 10.7554/eLife.31311 PMC5648532 28984244

[58] Wang X (2015). N(6)-methyladenosine modulates messenger RNA translation efficiency. Cell.

[59] Alarcón CR (2015). N6-methyladenosine marks primary microRNAs for processing. Nature.

[60] Wu B (2018). Molecular basis for the specific and multivariant recognitions of RNA substrates by human hnRNP A2/B1. Nat. Commun..

[61] Shi H (2017). YTHDF3 facilitates translation and decay of N-methyladenosine-modified RNA. Cell Res..

[62] Hsu PJ (2017). Ythdc2 is an N-methyladenosine binding protein that regulates mammalian spermatogenesis. Cell Res..

[63] Bell JL (2013). Insulin-like growth factor 2 mRNA-binding proteins (IGF2BPs): post-transcriptional drivers of cancer progression?. Cell. Mol. life Sci..

[64] Huang H (2018). Recognition of RNA N-methyladenosine by IGF2BP proteins enhances mRNA stability and translation. Nat. Cell Biol..

[65] Liu J, Harada BT, He C (2019). Regulation of gene expression by N(6)-methyladenosine in cancer. Trends Cell Biol..

[66] Palis J (2014). Primitive and definitive erythropoiesis in mammals. Front. Physiol..

[67] Yoder MC (2014). Inducing definitive hematopoiesis in a dish. Nat. Biotechnol..

[68] Potts KS (2014). A lineage of diploid platelet-forming cells precedes polyploid megakaryocyte formation in the mouse embryo. Blood.

[69] Kingsley PD, Malik J, Fantauzzo KA, Palis J (2004). Yolk sac-derived primitive erythroblasts enucleate during mammalian embryogenesis. Blood.

[70] Van Handel B (2010). The first trimester human placenta is a site for terminal maturation of primitive erythroid cells. Blood.

[71] Gomez Perdiguero E (2015). Tissue-resident macrophages originate from yolk-sac-derived erythro-myeloid progenitors. Nature.

[72] McGrath KE (2015). Distinct sources of hematopoietic progenitors emerge before HSCs and provide functional blood cells in the mammalian embryo. Cell Rep..

[73] Kiel MJ (2007). Haematopoietic stem cells do not asymmetrically segregate chromosomes or retain BrdU. Nature.

[74] Kunimoto H, Nakajima H (2017). Epigenetic dysregulation of hematopoietic stem cells and preleukemic state. Int. J. Hematol..

[75] Raghuwanshi S (2018). Epigenetic mechanisms: role in hematopoietic stem cell lineage commitment and differentiation. Curr. Drug Targets.

[76] Gore AV, Weinstein BM (2016). DNA methylation in hematopoietic development and disease. Exp. Hematol..

[77] Krivtsov AV, Armstrong SA (2007). MLL translocations, histone modifications and leukaemia stem-cell development. Nat. Rev. Cancer.

[78] Cerny J, Quesenberry PJ (2004). Chromatin remodeling and stem cell theory of relativity. J. Cell Physiol..

[79] Rodríguez-Malavé NI, Rao DS (2016). Long noncoding RNAs in hematopoietic malignancies. Brief. Funct. Genomics..

[80] Vu LP, Cheng Y, Kharas MG (2019). The biology of m(6)A RNA methylation in normal and malignant hematopoiesis. Cancer Discov..

[81] Weng H, Huang H, Chen J (2019). RNA N (6)-methyladenosine modification in normal and malignant hematopoiesis. Adv. Exp. Med. Biol..

[82] Hsu PJ, He C (2017). Making changes: N(6)-methyladenosine-mediated decay drives the endothelial-to-hematopoietic transition. Biochemistry.

[83] Zhang C, Liu F (2018). RNA methylation regulates hematopoietic stem/progenitor cell specification. Sci. China Life Sci..

[84] Zhang C (2017). m(6)A modulates haematopoietic stem and progenitor cell specification. Nature.

[85] Bonaguidi MA (2011). In vivo clonal analysis reveals self-renewing and multipotent adult neural stem cell characteristics. Cell.

[86] Price J, Thurlow L (1988). Cell lineage in the rat cerebral cortex: a study using retroviral-mediated gene transfer. Development..

[87] Turner DL, Cepko CL (1987). A common progenitor for neurons and glia persists in rat retina late in development. Nature.

[88] Walsh C, Cepko CL (1992). Widespread dispersion of neuronal clones across functional regions of the cerebral cortex. Science.

[89] Kornack DR, Rakic P (1995). Radial and horizontal deployment of clonally related cells in the primate neocortex: relationship to distinct mitotic lineages. Neuron.

[90] Englund C (2005). Pax6, Tbr2, and Tbr1 are expressed sequentially by radial glia, intermediate progenitor cells, and postmitotic neurons in developing neocortex. J. Neurosci..

[91] Anderson DJ (1989). The neural crest cell lineage problem: neuropoiesis?. Neuron.

[92] Cattaneo E, McKay R (1990). Proliferation and differentiation of neuronal stem cells regulated by nerve growth factor. Nature.

[93] Davis AA, Temple S (1994). A self-renewing multipotential stem cell in embryonic rat cerebral cortex. Nature.

[94] Kilpatrick TJ, Bartlett PF (1993). Cloning and growth of multipotential neural precursors: requirements for proliferation and differentiation. Neuron.

[95] Temple S (1989). Division and differentiation of isolated CNS blast cells in microculture. Nature.

[96] Kempermann G, Gage FH (1999). New nerve cells for the adult brain. Sci. Am..

[97] Reynolds BA, Weiss S (1992). Generation of neurons and astrocytes from isolated cells of the adult mammalian central nervous system. Science.

[98] Ma DK (2010). Epigenetic choreographers of neurogenesis in the adult mammalian brain. Nat. Neurosci..

[99] Liu C (2010). Epigenetic regulation of miR-184 by MBD1 governs neural stem cell proliferation and differentiation. Cell. Stem Cell..

[100] Li X, Jin P (2010). Roles of small regulatory RNAs in determining neuronal identity. Nat. Rev. Neurosci..

[101] Livneh I (2020). The m(6)A epitranscriptome: transcriptome plasticity in brain development and function. Nat. Rev. Neurosci..

[102] Li J (2019). The role of mRNA m(6)A methylation in the nervous system. Cell Biosci..

[103] Du K, Zhang L, Lee T, Sun T (2019). m(6)A RNA methylation controls neural development and is involved in human diseases. Mol. Neurobiol..

[104] Wang CX (2018). METTL3-mediated m6A modification is required for cerebellar development. PLoS Biol..

[105] Zhuang M (2019). The m6A reader YTHDF1 regulates axon guidance through translational control of Robo3.1 expression. Nucleic Acids Res..

[106] Yoon KJ (2017). Temporal control of mammalian cortical neurogenesis by m(6)A methylation. Cell.

[107] Saitou M, Miyauchi H (2016). Gametogenesis from pluripotent stem cells. Cell. Stem Cell..

[108] Ginsburg M, Snow MH, McLaren A (1990). Primordial germ cells in the mouse embryo during gastrulation. Development.

[109] Saitou M, Barton SC, Surani MA (2002). A molecular programme for the specification of germ cell fate in mice. Nature.

[110] Culty M (2009). Gonocytes, the forgotten cells of the germ cell lineage. Birth Defects Res. C.

[111] Molyneaux KA, Stallock J, Schaible K, Wylie C (2001). Time-lapse analysis of living mouse germ cell migration. Dev. Biol..

[112] Seki Y (2007). Cellular dynamics associated with the genome-wide epigenetic reprogramming in migrating primordial germ cells in mice. Development.

[113] Tam PP, Snow MH (1981). Proliferation and migration of primordial germ cells during compensatory growth in mouse embryos. J. Embryol. Exp. Morphol..

[114] Hilscher B (1974). Kinetics of gametogenesis. I. Comparative histological and autoradiographic studies of oocytes and transitional prospermatogonia during oogenesis and prespermatogenesis. Cell Tissue Res..

[115] Speed RM (1982). Meiosis in the foetal mouse ovary. I. An analysis at the light microscope level using surface-spreading. Chromosoma.

[116] Lee HJ, Hore TA, Reik W (2014). Reprogramming the methylome: erasing memory and creating diversity. Cell. Stem Cell..

[117] Saitou M, Kagiwada S, Kurimoto K (2012). Epigenetic reprogramming in mouse pre-implantation development and primordial germ cells. Development.

[118] Lin Z, Tong MH (2019). m(6)A mRNA modification regulates mammalian spermatogenesis. Biochim. Biophys. Acta Gene Regulatory Mech.

[119] Hsu PJ (2017). Ythdc2 is an N(6)-methyladenosine binding protein that regulates mammalian spermatogenesis. Cell Res..

[120] Zhang Y, Gao S, Xia J, Liu F (2018). Hematopoietic hierarchy—an updated roadmap. Trends Cell Biol..

[121] Hart SM, Foroni L (2002). Core binding factor genes and human leukemia. Haematologica.

[122] Lv J (2018). Endothelial-specific m6A modulates mouse hematopoietic stem and progenitor cell development via Notch signaling. Cell Res..

[123] Vu LP (2017). The N(6)-methyladenosine (m(6)A)-forming enzyme METTL3 controls myeloid differentiation of normal hematopoietic and leukemia cells. Nat. Med..

[124] Wang H (2018). Loss of YTHDF2-mediated m(6)A-dependent mRNA clearance facilitates hematopoietic stem cell regeneration. Cell Res..

[125] Gao Y (2020). m(6)A modification prevents formation of endogenous double-stranded RNAs and deleterious innate immune responses during hematopoietic development. Immunity.

[126] Lence T (2016). mA modulates neuronal functions and sex determination in Drosophila. Nature.

[127] Li L (2017). Fat mass and obesity-associated (FTO) protein regulates adult neurogenesis. Hum. Mol. Genet..

[128] Yoon K-J (2017). Temporal control of mammalian cortical neurogenesis by mA methylation. Cell.

[129] Xu H (2020). m(6)A mRNA methylation is essential for oligodendrocyte maturation and CNS myelination. Neuron.

[130] Zhang Z (2018). METTL3-mediated N(6)-methyladenosine mRNA modification enhances long-term memory consolidation. Cell Res..

[131] Ma C (2018). RNA m(6)A methylation participates in regulation of postnatal development of the mouse cerebellum. Genome Biol..

[132] Lein ES (2007). Genome-wide atlas of gene expression in the adult mouse brain. Nature.

[133] Shi H (2018). m(6)A facilitates hippocampus-dependent learning and memory through YTHDF1. Nature.

[134] Weng Y-L (2018). Epitranscriptomic mA regulation of axon regeneration in the adult mammalian nervous system. Neuron.

[135] Li M (2018). Ythdf2-mediated m(6)A mRNA clearance modulates neural development in mice. Genome Biol..

[136] Merkurjev D (2018). Synaptic N(6)-methyladenosine (m(6)A) epitranscriptome reveals functional partitioning of localized transcripts. Nat. Neurosci..

[137] Wu R (2019). A novel m(6)A reader Prrc2a controls oligodendroglial specification and myelination. Cell Res..

[138] Ivanova I (2017). The RNA m(6)A reader YTHDF2 is essential for the post-transcriptional regulation of the maternal transcriptome and oocyte competence. Mol. Cell..

[139] Qi ST (2016). N6-methyladenosine sequencing highlights the involvement of mRNA methylation in oocyte meiotic maturation and embryo development by regulating translation in Xenopus Laevis. J. Biol. Chem..

[140] Xia H (2018). Mettl3 mutation disrupts gamete maturation and reduces fertility in Zebrafish. Genetics.

[141] Kasowitz SD (2018). Nuclear m6A reader YTHDC1 regulates alternative polyadenylation and splicing during mouse oocyte development. PLoS Genet..

[142] Hongay CF, Orr-Weaver TL (2011). Drosophila Inducer of MEiosis 4 (IME4) is required for Notch signaling during oogenesis. Proc. Natl Acad. Sci. USA.

[143] Wang X, Wang Y (2017). From histones to RNA: role of methylation in signal proteins involved in adipogenesis. Curr. protein Pept. Sci..

[144] Chen Q (2016). Fate decision of mesenchymal stem cells: adipocytes or osteoblasts?. Cell Death Differ..

[145] Chang, E. & Kim, C. Y. Natural products and obesity: a focus on the regulation of mitotic clonal expansion during adipogenesis. *Molecules*. **24**, 1157 (2019). 10.3390/molecules24061157 PMC6471203 30909556

[146] Claussnitzer M (2015). FTO obesity variant circuitry and adipocyte browning in humans. N. Engl. J. Med..

[147] Wang X (2020). m(6)A mRNA methylation controls autophagy and adipogenesis by targeting Atg5 and Atg7. Autophagy.

[148] Wu R (2018). FTO regulates adipogenesis by controlling cell cycle progression via m(6)A-YTHDF2 dependent mechanism. Biochim. Biophys. Acta Mol. Cell Biol. Lipids.

[149] Jiang Q (2019). MTCH2 promotes adipogenesis in intramuscular preadipocytes via an m(6)A-YTHDF1-dependent mechanism. FASEB J..

[150] Siang DTC (2020). The RNA-binding protein HuR is a negative regulator in adipogenesis. Nat. Commun..

[151] Yao Y (2019). METTL3 inhibits BMSC adipogenic differentiation by targeting the JAK1/STAT5/C/EBPβ pathway via an m(6)A-YTHDF2-dependent manner. FASEB J..

[152] Cen S (2020). TRAF4 acts as a fate checkpoint to regulate the adipogenic differentiation of MSCs by activating PKM2. EBioMedicine.

[153] Wu Y (2018). Mettl3-mediated m(6)A RNA methylation regulates the fate of bone marrow mesenchymal stem cells and osteoporosis. Nat. Commun..

[154] Yu J (2020). The m6A methyltransferase METTL3 cooperates with demethylase ALKBH5 to regulate osteogenic differentiation through NF-κB signaling. Mol. Cell. Biochem..

[155] Yan G (2020). m(6)A methylation of precursor-miR-320/RUNX2 controls osteogenic potential of bone marrow-derived mesenchymal stem cells. Mol. Ther. Nucleic Acids.

[156] Tian, C. et al. Mettl3 regulates osteogenic differentiation and alternative splicing of vegfa in bone marrow mesenchymal stem cells. *Int. J. Mol. Sci.* **20**, 551 (2019). 10.3390/ijms20030551 PMC6387109 30696066

[157] He S (2017). mRNA N6-methyladenosine methylation of postnatal liver development in pig. PLoS ONE.

[158] Huang H, Weng H, Chen J (2020). m(6)A modification in coding and non-coding RNAs: roles and therapeutic implications in cancer. Cancer Cell..

[159] Chen XY, Zhang J, Zhu JS (2019). The role of m(6)A RNA methylation in human cancer. Mol. Cancer.

[160] He L (2019). Functions of N6-methyladenosine and its role in cancer. Mol. Cancer.

[161] Hu BB (2019). N(6)-methyladenosine (m(6)A) RNA modification in gastrointestinal tract cancers: roles, mechanisms, and applications. Mol. Cancer.

[162] Melstrom L, Chen J (2020). RNA N(6)-methyladenosine modification in solid tumors: new therapeutic frontiers. Cancer Gene Ther..

[163] Boriack-Sjodin PA, Ribich S, Copeland RA (2018). RNA-modifying proteins as anticancer drug targets. Nat. Rev. Drug Discov..

[164] Wang S (2017). Roles of RNA methylation by means of N(6)-methyladenosine (m(6)A) in human cancers. Cancer Lett..

[165] Muñoz, M. & Coveñas, R. The Neurokinin-1 receptor antagonist aprepitant, a new drug for the treatment of hematological malignancies: focus on acute myeloid leukemia. *J. Clin. Med*. **9**, 1659 (2020). 10.3390/jcm9061659 PMC7355887 32492831

[166] Yang L, Han Y, Suarez Saiz F, Minden MD (2007). A tumor suppressor and oncogene: the WT1 story. Leukemia.

[167] Bansal H (2014). WTAP is a novel oncogenic protein in acute myeloid leukemia. Leukemia.

[168] Horiuchi K (2013). Identification of Wilms’ tumor 1-associating protein complex and its role in alternative splicing and the cell cycle. J. Biol. Chem..

[169] Raffel GD (2007). Ott1(Rbm15) has pleiotropic roles in hematopoietic development. Proc. Natl Acad. Sci. USA.

[170] Ma X (2007). Rbm15 modulates Notch-induced transcriptional activation and affects myeloid differentiation. Mol. Cell. Biol..

[171] Lv J (2018). Endothelial-specific m(6)A modulates mouse hematopoietic stem and progenitor cell development via Notch signaling. Cell Res..

[172] Vu LP (2017). The N-methyladenosine (mA)-forming enzyme METTL3 controls myeloid differentiation of normal hematopoietic and leukemia cells. Nat. Med..

[173] Weng H (2018). METTL14 inhibits hematopoietic stem/progenitor differentiation and promotes leukemogenesis via mRNA m(6)A modification. Cell. Stem Cell..

[174] Li Z (2017). FTO plays an oncogenic role in acute myeloid leukemia as a N(6)-methyladenosine RNA demethylase. Cancer Cell..

[175] Su R (2018). R-2HG exhibits anti-tumor activity by targeting FTO/m(6)A/MYC/CEBPA signaling. Cell.

[176] Yan F (2018). A dynamic N-methyladenosine methylome regulates intrinsic and acquired resistance to tyrosine kinase inhibitors. Cell Res..

[177] Shen C (2020). RNA demethylase ALKBH5 selectively promotes tumorigenesis and cancer stem cell self-renewal in acute myeloid leukemia. Cell. Stem Cell..

[178] Wang J (2020). Leukemogenic chromatin alterations promote AML leukemia stem cells via a KDM4C-ALKBH5-AXL signaling axis. Cell. Stem Cell..

[179] Paris J (2019). Targeting the RNA m(6)A reader YTHDF2 selectively compromises cancer stem cells in acute myeloid leukemia. Cell. Stem Cell..

[180] Elcheva IA (2020). RNA-binding protein IGF2BP1 maintains leukemia stem cell properties by regulating HOXB4, MYB, and ALDH1A1. Leukemia.

[181] Dai D, Wang H, Zhu L, Jin H, Wang X (2018). N6-methyladenosine links RNA metabolism to cancer progression. Cell Death Dis..

[182] Tan, A. C. et al. Management of glioblastoma: state of the art and future directions. *CA: Cancer J. Clin.*10.3322/caac.21613 (2020). 10.3322/caac.21613 32478924

[183] Chai R-C (2019). mA RNA methylation regulators contribute to malignant progression and have clinical prognostic impact in gliomas. Aging.

[184] Cui Q (2017). m(6)A RNA methylation regulates the self-renewal and tumorigenesis of glioblastoma stem cells. Cell Rep..

[185] Zhang S (2017). m(6)A demethylase ALKBH5 maintains tumorigenicity of glioblastoma stem-like cells by sustaining FOXM1 expression and cell proliferation program. Cancer Cell..

[186] Li F (2019). N(6)-methyladenosine modulates nonsense-mediated mRNA decay in human glioblastoma. Cancer Res..

[187] Jin DI (2012). Expression and roles of Wilms’ tumor 1-associating protein in glioblastoma. Cancer Sci..

[188] Babic I (2013). EGFR mutation-induced alternative splicing of Max contributes to growth of glycolytic tumors in brain cancer. Cell Metab..

[189] Deng J (2016). Effects of hnRNP A2/B1 knockdown on inhibition of glioblastoma cell invasion, growth and survival. Mol. Neurobiol..

[190] Brandi J (2016). The antioxidant uncoupling protein 2 stimulates hnRNPA2/B1, GLUT1 and PKM2 expression and sensitizes pancreas cancer cells to glycolysis inhibition. Free Radic. Biol. Med..

[191] Degrauwe N (2016). The RNA binding protein IMP2 preserves glioblastoma stem cells by preventing let-7 target gene silencing. Cell Rep..

[192] Janiszewska M (2012). Imp2 controls oxidative phosphorylation and is crucial for preserving glioblastoma cancer stem cells. Genes Dev..

[193] Wang R-J (2015). MicroRNA-873 (miRNA-873) inhibits glioblastoma tumorigenesis and metastasis by suppressing the expression of IGF2BP1. J. Biol. Chem..

[194] Yang Y (2020). Tumor suppressor microRNA-138 suppresses low-grade glioma development and metastasis via regulating IGF2BP2. OncoTargets Ther..

[195] Kouhkan F (2016). MicroRNA-129-1 acts as tumour suppressor and induces cell cycle arrest of GBM cancer cells through targeting IGF2BP3 and MAPK1. J. Med. Genet..

[196] Mineo M (2016). The long non-coding RNA HIF1A-AS2 Facilitates the maintenance of mesenchymal glioblastoma stem-like cells in hypoxic niches. Cell Rep..

[197] Jin P (2018). CircRNA circHIPK3 serves as a prognostic marker to promote glioma progression by regulating miR-654/IGF2BP3 signaling. Biochem. Biophys. Res. Commun..

[198] Han J (2019). METTL3 promote tumor proliferation of bladder cancer by accelerating pri-miR221/222 maturation in m6A-dependent manner. Mol. Cancer.

[199] Cheng M (2019). The m(6)A methyltransferase METTL3 promotes bladder cancer progression via AFF4/NF-κB/MYC signaling network. Oncogene.

[200] Xie H (2020). METTL3/YTHDF2 m(6) A axis promotes tumorigenesis by degrading SETD7 and KLF4 mRNAs in bladder cancer. J. Cell Mol. Med..

[201] Yang F (2019). Dynamic m(6)A mRNA methylation reveals the role of METTL3-m(6)A-CDCP1 signaling axis in chemical carcinogenesis. Oncogene.

[202] Gu C (2019). Mettl14 inhibits bladder TIC self-renewal and bladder tumorigenesis through N(6)-methyladenosine of Notch1. Mol. Cancer.

[203] Ding C (2018). Increased N6-methyladenosine causes infertility is associated with FTO expression. J. Cell Physiol..

[204] Zhu H (2019). ALKBH5 inhibited autophagy of epithelial ovarian cancer through miR-7 and BCL-2. J. Exp. Clin. Cancer Res..

[205] Jiang Y (2020). RNA demethylase ALKBH5 promotes ovarian carcinogenesis in a simulated tumour microenvironment through stimulating NF-κB pathway. J. Cell Mol. Med..

[206] Yang Y (2020). Loss of hnRNPA2B1 inhibits malignant capability and promotes apoptosis via down-regulating Lin28B expression in ovarian cancer. Cancer Lett..

[207] Müller S (2019). IGF2BP1 promotes SRF-dependent transcription in cancer in a m6A- and miRNA-dependent manner. Nucleic Acids Res..

[208] Liu H (2019). Overexpression of IGF2BP3 as a potential oncogene in ovarian clear cell carcinoma. Front. Oncol..

[209] Wang X (2017). Reduced m(6)A mRNA methylation is correlated with the progression of human cervical cancer. Oncotarget.

[210] Zhou S (2018). FTO regulates the chemo-radiotherapy resistance of cervical squamous cell carcinoma (CSCC) by targeting β-catenin through mRNA demethylation. Mol. Carcinog..

[211] Ma XX, Cao ZG, Zhao SL (2020). m6A methyltransferase METTL3 promotes the progression of prostate cancer via m6A-modified LEF1. Eur. Rev. Med Pharm. Sci..

[212] Yuan Y, Du Y, Wang L, Liu X (2020). The M6A methyltransferase METTL3 promotes the development and progression of prostate carcinoma via mediating MYC methylation. J. Cancer.

[213] Engel M (2018). The role of m(6)A/m-RNA methylation in stress response regulation. Neuron.

[214] Liu X (2016). Genome-wide association study of autism spectrum disorder in the east asian populations. Autism Res..

[215] Oldmeadow C (2014). Combined analysis of exon splicing and genome wide polymorphism data predict schizophrenia risk loci. J. Psychiatr. Res..

[216] Keller L (2011). The obesity related gene, FTO, interacts with APOE, and is associated with Alzheimer’s disease risk: a prospective cohort study. J. Alzheimer’s Dis..

[217] Han M (2020). Abnormality of m6A mRNA methylation is involved in Alzheimer’s disease. Front. Neurosci..

[218] Huang R (2020). N(6)-methyladenosine modification of fatty acid amide hydrolase messenger RNA in circular RNA STAG1-regulated astrocyte dysfunction and depressive-like behaviors. Biol. Psychiatry.

[219] Liu Q (2019). ZFP217 regulates adipogenesis by controlling mitotic clonal expansion in a METTL3-m(6)A dependent manner. RNA Biol..

[220] Xie W (2019). METTL3 inhibits hepatic insulin sensitivity via N6-methyladenosine modification of Fasn mRNA and promoting fatty acid metabolism. Biochem. Biophys. Res. Commun..

[221] Li, Y. et al. m(6)A Regulates liver metabolic disorders and hepatogenous diabetes. *Genomics Proteomics Bioinform.* (2020). 10.1016/j.gpb.2020.06.003 PMC8242261 33160098

[222] Zhou, B. et al. N(6)-methyladenosine reader protein YT521-B Homology domain-containing 2 suppresses liver steatosis by regulation of mRNA stability of lipogenic genes. *Hepatology*10.1002/hep.31220 (2020). 10.1002/hep.31220 32150756

[223] De Jesus DF (2019). m(6)A mRNA methylation regulates human ?2-cell biology in physiological states and in type 2 diabetes. Nat. Metab..

[224] Qin Y (2020). Role of m6A RNA methylation in cardiovascular disease (review). Int J. Mol. Med..

[225] Zhao K (2020). Epigenetic role of N6-methyladenosine (m6A) RNA methylation in the cardiovascular system. J. Zhejiang Univ. Sci. B..

[226] Dorn LE (2019). The N(6)-methyladenosine mRNA methylase METTL3 controls cardiac homeostasis and hypertrophy. Circulation.

[227] Mathiyalagan P (2019). FTO-dependent N(6)-methyladenosine regulates cardiac function during remodeling and repair. Circulation.

[228] Song H (2019). METTL3 and ALKBH5 oppositely regulate m(6)A modification of TFEB mRNA, which dictates the fate of hypoxia/reoxygenation-treated cardiomyocytes. Autophagy.

[229] Chen J (2019). METTL14-dependent m6A regulates vascular calcification induced by indoxyl sulfate. Life Sci..

[230] Zhu B (2020). Total Panax notoginseng saponin inhibits vascular smooth muscle cell proliferation and migration and intimal hyperplasia by regulating WTAP/p16 signals via m(6)A modulation. Biomed. Pharmacother..

[231] Li A (2017). Cytoplasmic m(6)A reader YTHDF3 promotes mRNA translation. Cell Res..

[232] Shi H (2017). YTHDF3 facilitates translation and decay of N(6)-methyladenosine-modified RNA. Cell Res..

[233] Zaccara S, Jaffrey SR (2020). A unified model for the function of YTHDF proteins in regulating m(6)A-modified mRNA. Cell.

[234] Visvanathan A (2018). Essential role of METTL3-mediated m(6)A modification in glioma stem-like cells maintenance and radioresistance. Oncogene.

[235] Dominissini D (2013). Transcriptome-wide mapping of N(6)-methyladenosine by m(6)A-seq based on immunocapturing and massively parallel sequencing. Nat. Protoc..

[236] Tuck MT, James CB, Kelder B, Kopchick JJ (1996). Elevation of internal 6-methyladenine mRNA methyltransferase activity after cellular transformation. Cancer Lett..

[237] Zhang C (2016). Hypoxia-inducible factors regulate pluripotency factor expression by ZNF217- and ALKBH5-mediated modulation of RNA methylation in breast cancer cells. Oncotarget.

[238] Lin S (2016). The m(6)A methyltransferase METTL3 promotes translation in human cancer cells. Mol. cell..

[239] Liu N (2015). N(6)-methyladenosine-dependent RNA structural switches regulate RNA-protein interactions. Nature.

[240] Kwok CT, Marshall AD, Rasko JE, Wong JJ (2017). Genetic alterations of m(6)A regulators predict poorer survival in acute myeloid leukemia. J. Hematol. Oncol..

[241] Xiang Y (2017). RNA m(6)A methylation regulates the ultraviolet-induced DNA damage response. Nature.

[242] Yi C, Pan T (2011). Cellular dynamics of RNA modification. Acc. Chem. Res..

[243] Batista PJ (2014). m(6)A RNA modification controls cell fate transition in mammalian embryonic stem cells. Cell. Stem Cell..

[244] Chen T (2015). m(6)A RNA methylation is regulated by microRNAs and promotes reprogramming to pluripotency. Cell. Stem Cell..

[245] Geula S (2015). Stem cells. m6A mRNA methylation facilitates resolution of naive pluripotency toward differentiation. Science.

[246] Burger J (2020). Treatment of chronic lymphocytic leukemia. N. Engl. J. Med..

[247] Su R (2018). R-2HG exhibits anti-tumor activity by targeting FTO/mA/MYC/CEBPA signaling. Cell.

[248] Huang Y (2015). Meclofenamic acid selectively inhibits FTO demethylation of m6A over ALKBH5. Nucleic Acids Res..

[249] He W (2015). Identification of A novel small-molecule binding site of the fat mass and obesity associated protein (FTO). J. Med. Chem..

[250] Huang Y (2019). Small-molecule targeting of oncogenic FTO demethylase in acute myeloid leukemia. Cancer cell..

[251] Su, R. et al. Targeting FTO suppresses cancer stem cell maintenance and immune evasion. *Cancer Cell* **38**, 79–96.e11 (2020). 10.1016/j.ccell.2020.04.017 PMC7363590 32531268

[252] Singh B (2016). Important role of FTO in the survival of rare panresistant triple-negative inflammatory breast cancer cells facing a severe metabolic challenge. PLoS ONE.

[253] Li, J. et al. Targeted mRNA demethylation using an engineered dCas13b-ALKBH5 fusion protein. *Nucleic Acids Res.* **48**, 5684–5694 (2020). 10.1093/nar/gkaa269 PMC7261189 32356894

[254] Wang H (2019). Mettl3-mediated mRNA m(6)A methylation promotes dendritic cell activation. Nat. Commun..

[255] Han D (2019). Anti-tumour immunity controlled through mRNA m(6)A methylation and YTHDF1 in dendritic cells. Nature.

[256] Yang S (2019). m(6)A mRNA demethylase FTO regulates melanoma tumorigenicity and response to anti-PD-1 blockade. Nat. Commun..

[257] Dixit, D. et al. The RNA m6A reader YTHDF2 maintains oncogene expression and is a targetable dependency in glioblastoma stem cells. *Cancer Discov.*10.1158/2159-8290.cd-20-0331 (2020). 10.1158/2159-8290.CD-20-0331 PMC8110214 33023892

